# Natural selection of a *GSK3* determines rice mesocotyl domestication by coordinating strigolactone and brassinosteroid signaling

**DOI:** 10.1038/s41467-018-04952-9

**Published:** 2018-06-28

**Authors:** Shiyong Sun, Tao Wang, Linlin Wang, Xiaoming Li, Yancui Jia, Chang Liu, Xuehui Huang, Weibo Xie, Xuelu Wang

**Affiliations:** 1 National Key Laboratory of Crop Genetic Improvement, Center of Integrative Biology, College of Life Science and Technology, Huazhong Agricultural University, Wuhan, China; 2 Department of Genetics, School of Life Sciences, Fudan University, Shanghai, China; 3 College of Life and Environment Sciences, Shanghai Normal University, Shanghai, China; 4 National Key Laboratory of Crop Genetic Improvement, National Center of Plant Gene Research, Huazhong Agricultural University, Wuhan, China

## Abstract

Mesocotyl is the crucial organ for pushing buds out of deep water or soil after germination in monocots. Deep direct seeding or mechanized dry seeding cultivation practice requires rice cultivars having long mesocotyl. However, the mechanisms of mesocotyl elongation and domestication remain unknown. Here, our genome-wide association study (GWAS) reveals that natural variations of *OsGSK2*, a conserved *GSK3*-like kinase involved in brassinosteroid signaling, determine rice mesocotyl length variation. Variations in the coding region of *OsGSK2* alter its kinase activity. It is selected for mesocotyl length variation during domestication. Molecular analyses show that brassinosteroid-promoted mesocotyl elongation functions by suppressing the phosphorylation of an U-type cyclin, CYC U2, by OsGSK2. Importantly, the F-box protein D3, a major positive component in strigolactone signaling, can degrade the OsGSK2-phosphorylated CYC U2 to inhibit mesocotyl elongation. Together, these results suggest that *OsGSK2* is selected to regulate mesocotyl length by coordinating strigolactone and brassinosteroid signaling during domestication.

## Abstract (web summary)

Long mesocotyl is a critical trait for the application of rice deep direct seeding or mechanized dry seeding cultivation method. Here, Sun et al. find *OsGSK2* is selected for mesocotyl length variation during domestication and it coordinates strigolactone and brassinosteroid signaling to determine mesocotyl elongation.

## Introduction

Rice (*Oryza sativa*) is one of the most important cereal crops grown worldwide, indicating that a large diversity of genetic and important traits have been selected to help rice adapt to local environments or human cultivation practices^1^. In recent years, the deep direct seeding and mechanized dry seeding, the less cost cultivation modes, are imminently needed in rice-grown region to relieve the conflicts raised by the shortage of water, land, labor, and energy, which are required by traditional transplanting seedling system in preparing the field, uprooting, and transplanting the seedlings^2,3^. The mesocotyl elongation largely determines the successful seedling establishment under the less cost cultivation modes. The mesocotyl, an organ between the coleoptilar node and the basal part of the seminal root in young monocot seedlings, plays a key role in pushing buds out of the deep water or soil during germination for successful seedling establishment^4–6^. The ability of mesocotyl elongation exhibits high diversity among wild rice, weedy rice, and cultivated rice, but few of cultivated rice grown via transplanting seedling system has long mesocotyl (>1.0 cm)^7,8^, indicating that mesocotyl length may be selected during the traditional cultivation. However, the domestic and molecular mechanisms evolved for regulating rice mesocotyl length variation are poorly understood.

Mesocotyl elongation is highly regulated by various phytohormones^9–13^. Strigolactones (SLs), a recently identified class of terpenoid phytohormones, regulate many aspects of plant development, including shoot branching, germination, and mesocotyl length. SLs are perceived by the receptor D14 (Dwarf 14), and trigger an interaction between the D14 and D3 (Dwarf 3) and degradation of their targets through D3 ^14,15^. *D3*, encoding an F-box leucine-rich repeat protein, is a subunit of the Skp–Cullin–F-box (SCF) E3 ubiquitin ligase complex and is responsible for recruiting substrates of the SCF complex for ubiquitination^16^. The targets of D3, including D53 in rice and BES1 in *Arabidopsis*, undergo an SL-induced protein degradation, which is required for SL signaling^14,15,17^. It has been reported that the SL-inhibited rice mesocotyl elongation occurs through cell division, which is dependent on the SL signaling components D14 and D3 ^9^. However, the underlying molecular mechanism is currently unknown.

Brassinosteroids (BRs), a class of plant-specific steroid hormones, play key roles in regulating many aspects of plant development^18^. In rice, when the BR receptor OsBRI1 binds to BRs, BR signaling is activated, which inhibits the kinase activity of OsGSK2, a conserved glycogen synthase kinase 3 (GSK3)-like kinase, and results in reducing OsGSK2’s ability for phosphorylating its targets and enhancing BR signaling^19,20^. However, whether and/or how BR signaling regulates mesocotyl elongation is still unknown. Especially, how the mesocotyl elongation integrates the different signals for successful seedling establishment is worth being uncovered.

Here, we perform a genome-wide association study (GWAS) for rice mesocotyl length and demonstrate that natural alleles of *OsGSK2* in coding regions with the different kinase activities address mesocotyl variation and domestication from *Oryza rufipogon* to the cultivated rice; BRs promote mesocotyl elongation via cell division controlled by CYC U2, a plant-specific U-type cyclin, and OsGSK2 can phosphorylate CYC U2 to reduce its protein stability. Furthermore, we demonstrate that SLs inhibit mesocotyl elongation by degrading OsGSK2-phosphorylated CYC U2 by D3. Therefore, our findings not only reveal an important mechanism in mesocotyl elongation coordinately controlled by SL and BR signaling, but also indicate that *OsGSK2* is a key locus under the selection for mesocotyl length variation during rice domestication.

## Results

### *OsGSK2* determines mesocotyl variation and domestication

**Fig. 1:**
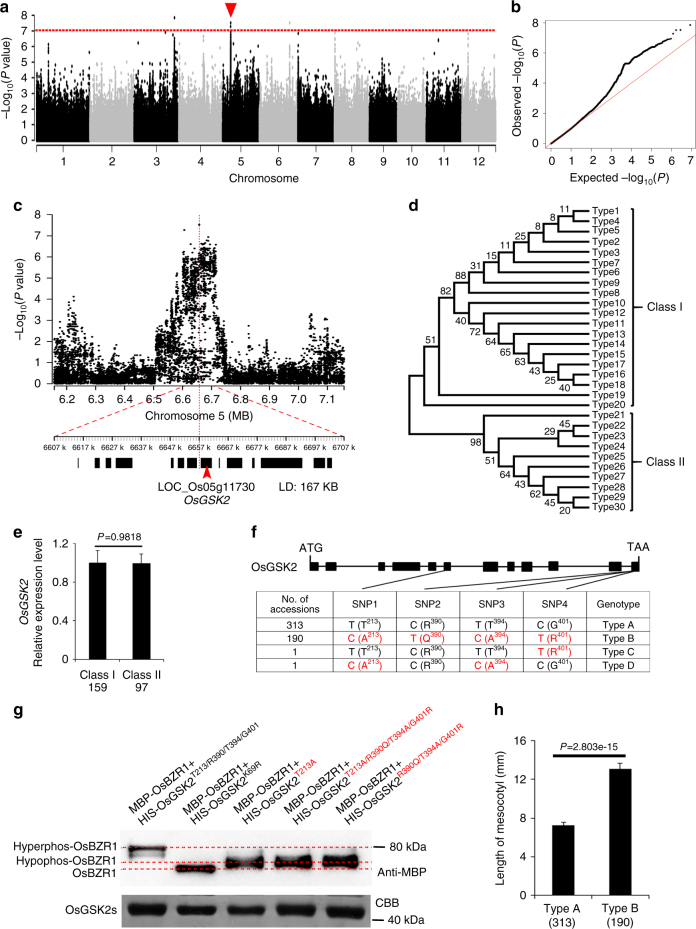
Genome-wide association study for mesocotyl length variation in rice. **a** Manhattan plots for mesocotyl length using a linear mixed model (LMM) in 510 *O. sativa* accessions. The red horizontal dashed line indicates the genome-wide significant threshold (*P* = 1.19 × 10^−7^). The red triangle shows the lead SNP association with *P* values below 1.19 × 10^−7^ on Chr5. **b** Quantile–quantile plot for mesocotyl length in 510 *O. sativa* populations. **c** Regions of the genome showing association signals with the lead SNP on chromosome 5 indicated in **a**. Top of the panel shows the region on each side of the lead SNP (SNP with the lowest *P* value), whose position is indicated by a vertical red line. *P* values from the LMM are plotted on the *y*-axis. The bottom of the panel shows a 50-kb region on each side of the lead SNP, with the candidate genes indicated by black boxes. Local LD of the chromosomal regions containing lead SNP is given (LD: 167 kb). **d** Phylogenetic tree of the 30 haplotypes of the *OsGSK2* promoter regions constructed using the neighbor-jointing method. Branch length represents the number of observed polymorphisms on the branch. **e** The relative transcriptional levels of *OsGSK2* of cultivars in class I and class II, respectively. The number of accessions analyzed is shown below each bar. Error bars are SD (*n* = 3). **f** Structure of the coding region of *OsGSK2* and DNA polymorphism in this gene. The four nonsynonymous SNPs in the OsGSK2 coding region are indicated. The one-letter amino acid codes related to the four nonsynonymous SNPs are showed in the brackets with the loci in OsGSK2 protein. **g** The phosphorylation activity of different forms of OsGSK2 in vitro. The phosphorylation status of MBP-OsBZR1 was detected by anti-MBP antibody. We have independently repeated this assay for three times. **h** Analysis of mesocotyl length in the accessions with the indicated genotypes of *OsGSK2*. The 313 accessions with *OsGSK2*^Type A^ and 190 accessions with *OsGSK2*^Type B^ are included in this analysis. Error bars are SE. *P* value is determined by Welch’s two-sample *t* test

To discover genes regulating mesocotyl elongation and domestication, we applied a GWAS for mesocotyl length among a diverse worldwide collection of 510 *O. sativa* accessions using a linear mixed model (LMM) (Supplementary Data 1). We identified three significantly associated loci meeting a suggestive threshold (*P* < 1.19 × 10^−7^; false discovery rate <0.01) (Fig. 1a, b and Supplementary Table 1). Interestingly, we found that *OsGSK2*, the key negative component in the BR signaling^19^, is immediately adjacent to the lead single-nucleotide polymorphism (SNP) in a major associated locus on chromosome 5 (*P* = 3 × 10^−8^) and is within an 87 bp distance from the lead SNP of this associated locus, suggesting that *OsGSK2* is a strong candidate gene in this associated locus (Fig. 1c and Supplementary Table 2).

To identify SNPs in *OsGSK2* associated with the natural variation in mesocotyl length, we first analyzed the SNPs in the *OsGSK2* promoter regions (2.0 kb region upstream of the translation start site) from 504 rice accessions. We divided the sequences into 30 haplotypes, which were classified into two classes by phylogenetic analysis: types 1–20 in class I and types 21–30 in class II (Fig. 1d). There were three consensus SNPs that could differentiate class I from class II (at −1412, −1632, and −1637) (Supplementary Fig. 1). To investigate their effects on *OsGSK2* expression, we randomly selected 159 accessions in class I and 97 accessions in class II, and measured the expression levels of *OsGSK2* in mesocotyls by quantitative reverse transcription PCR (RT-qPCR) (Supplementary Data 2). However, we did not find significant difference in its expression levels between the two classes (Fig. 1e), suggesting that the variation in the promoter regions of *OsGSK2* are not associated with the natural variation of mesocotyl length. Furthermore, we found four nonsynonymous SNPs in the *OsGSK2* coding region (Fig. 1f). Based on these four SNPs, we divided the sequences of the 505 cultivated varieties into four haplotypes, including 313 accessions in type A, 190 accessions in type B, 1 accession in type C, and 1 accession in type D (Fig. 1f). In BR signaling pathway, the activated BR signaling inhibits the kinase activity of OsGSK2/BIN2, resulting in altering the phosphorylation status of BZR1/BES1 to transduce BR signaling^21,22^. The different phosphorylation levels in BZR1/BES1 can be distinguished by different protein sizes using western blot^22,23^, which has been widely used to evaluate the kinase activity of OsGSK2/BIN2 (ref. 24). To determine whether these haplotypes affect OsGSK2 activity, we conducted in vitro kinase assays using OsBZR1 as their target, the known substrates of OsGSK2 in the BR signaling^20,22^, and we found that the hyperphos-BZR1 with the larger protein size was detected in the OsGSK2^typeA^ kinase assay, the hypophos-BZR1 with the smaller protein sizes (the intermediate band sizes) was detected in the OsGSK2^typeB^/OsGSK2^typeB^-like kinase assay, and the BZR1 in the OsGSK2^K69R^ kinase assay, a kinase dead form similar to AtBIN2^K69R^, which eliminated GSK/SHAGGY kinase activity^19^ (Fig. 1g), is as a negative control, which suggested that the kinase activity of OsGSK2^typeB^/OsGSK2^typeB^-like is much weaker than that of OsGSK2^typeA^ and only slightly higher than that of OsGSK2^K69R^. These results suggested that the four nonsynonymous SNPs are important for determining the kinase activity of OsGSK2. More importantly, cultivars carrying the *OsGSK2*^*typeA*^ allele (with high kinase activity) tended to have much shorter mesocotyls than those harboring the *OsGSK2*^*typeB*^ allele (with low kinase activity) (Fig. 1h).

**Fig. 2:**
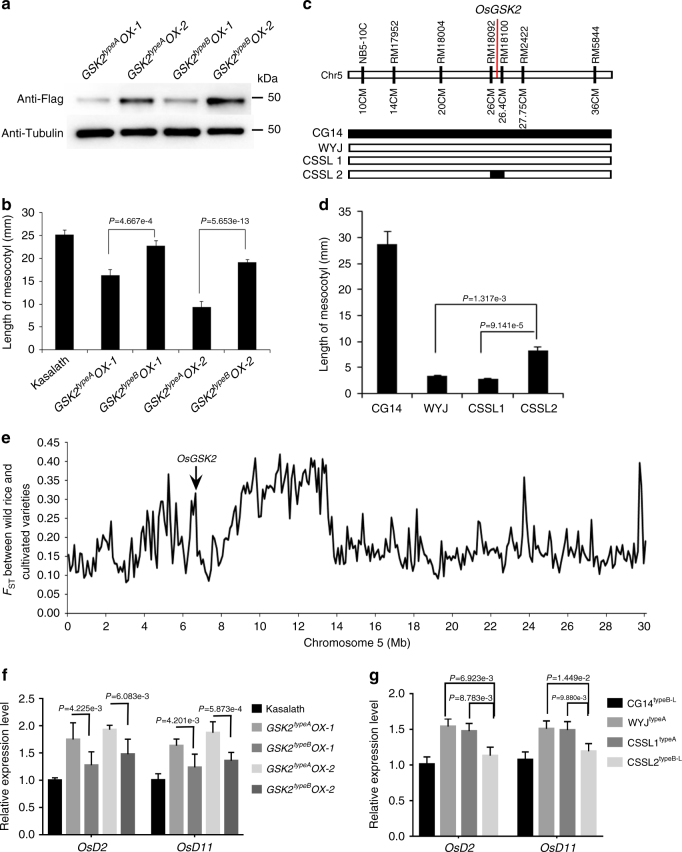
The natural alleles of *OsGSK2* function in mesocotyl length and BR signaling. **a** The protein levels of OsGSK2^typeA^-FLAG and OsGSK2^typeB^-FLAG in the independent transgenic rice in Kasalath background. **b** Mesocotyl length of *OsGSK2*^*typeA*^ and *OsGSK2*^*typeB*^ transgenic rice in **a**. Kasalath is the background. Error bars are SE (*n* = 30). *P* value is determined by Welch’s *t* test with Bonferroni correction. **c** Graphical genotypes of the chromosome segment substitution lines used in this study. WYJ is the recurrent parent and CG14 is the donor parent. CSSL1 and CSSL2 are two substitution lines. CSSL1 is a control segregated from the CSSLs population. **d** The mesocotyl length of the CSSLs and their parents indicated in **c**. Error bars are SE (*n* = 25). *P* value is determined by Welch’s *t* test with Bonferroni correction. **e** The level of genetic differentiation (*F*_ST_) across chromosome 5 between the *Oryza rufipogon* and cultivated rice. The *F*_ST_ level of *OsGSK2* locus is 0.316, and the average *F*_ST_ level in the whole genome is 0.172. **f**–**g** The relative transcript levels of *OsD2* and *OsD11* in *OsGSK2* transgenic lines from **a** and in the CSSLs from **c**. Total RNAs were extracted from the mesocotyls. The transcript level in WT was defined as “1”. Data are means ± SD (*n* = 3). *P* value is determined by Welch’s *t* test with Bonferroni correction

To further demonstrate whether the difference in activities of the two major alleles of *OsGSK2* is responsible for difference in mesocotyl elongation in planta, we generated transgenic rice with *OsGSK2*^*typeA*^*-FLAG* and *OsGSK2*^*typeB*^*-FLAG* in Kasalath background, which harbors the endogenous *OsGSK2*^*typeB*^ allele. The *OsGSK2*^*typeB*^*-FLAG* transgenic rice exhibited significantly longer mesocotyls than the *OsGSK2*^*typeA*^*-FLAG* transgenic rice (Fig. 2a, b), indicating that the activity of OsGSK2^typeA^ is higher than that of OsGSK2^typeB^ to inhibit rice mesocotyl elongation. We further evaluated the mesocotyl length of rice germplasms with the different *OsGSK2* alleles using the chromosome single-segment substitution lines (CSSLs). We obtained the CSSLs with Wuyunjing (WYJ; *O. sativa* ssp. *japonica*) as the recurrent parent and CG14 (an *O. glaberrima* accession) as the donor parent, and found that the recurrent parent WYJ with endogenous *OsGSK2*^*typeA*^ allele had shorter mesocotyls than the donor parent CG14 with an endogenous allele type similar to *OsGSK2*^*typeB*^ (including the SNPs 637 and 1180; Supplementary Fig. 2a) (Fig. 2c, d). In addition, the mesocotyls of CSSL2, which carries a substituted segment on chromosome 5 from CG14 in the WYJ background, were longer than those of CSSL1 (a control line segregated from the CSSL population) and the recurrent parent WYJ (Fig. 2c, d). Taken together, we concluded that the *OsGSK2*^*typeA*^ allele with high kinase activity results in short mesocotyl in rice.

To determine the domesticated origins of the different alleles, we analyzed the *OsGSK2* orthologs from the wild AA-genome species^25^ and found that the *OsGSK2*^*typeA*^ allele in cultivated rice was derived from *O. rufipogon*, the direct ancestor of Asian cultivated rice (Supplementary Fig. 2b). Further assessment of the *OsGSK2* orthologs in the 397 *O. rufipogon*^26^ and the 947 cultivated rice^27^ showed that 351 *O. rufipogon* accessions contained *OsGSK2*^*typeB*^ or *OsGSK2*^*typeB*^*-like* alleles, with weak kinase activity, whereas only 40 *O. rufipogon* accessions contained the *OsGSK2*^*typeA*^ allele (Supplementary Fig. 2c). However, among the 947 cultivated rice accessions, only 233 accessions contained the *OsGSK2*^*typeB*^ or *OsGSK2*^*typeB*^-like alleles, while 714 accessions contained the *OsGSK2*^*typeA*^ allele (Supplementary Fig. 2d), indicating that the allelic frequency of *OsGSK2* was directionally selected during the domestication from the *O. rufipogon* to the cultivated rice. Moreover, we estimated the domestication of *OsGSK2* by detecting the level of population difference (*F*_ST_) at the whole genome level between the *O. rufipogon* and the cultivated rice, and found that the *F*_ST_ level in the *OsGSK2* locus was 0.316, which is significantly higher than the average *F*_ST_ level in the whole genome (0.172) (Fig. 2e). We further calculated the percentile ranking of *OsGSK2* *F*_ST_ values and found that it was at the 95.5th percentile among all genes in the whole genome. Together, the results suggested that the different alleles of *OsGSK2* have undergone directional selection during the domestication from the *O. rufipogon* to the cultivated rice.

### *CYC U2* is required for BR-regulated mesocotyl elongation

**Fig. 3:**
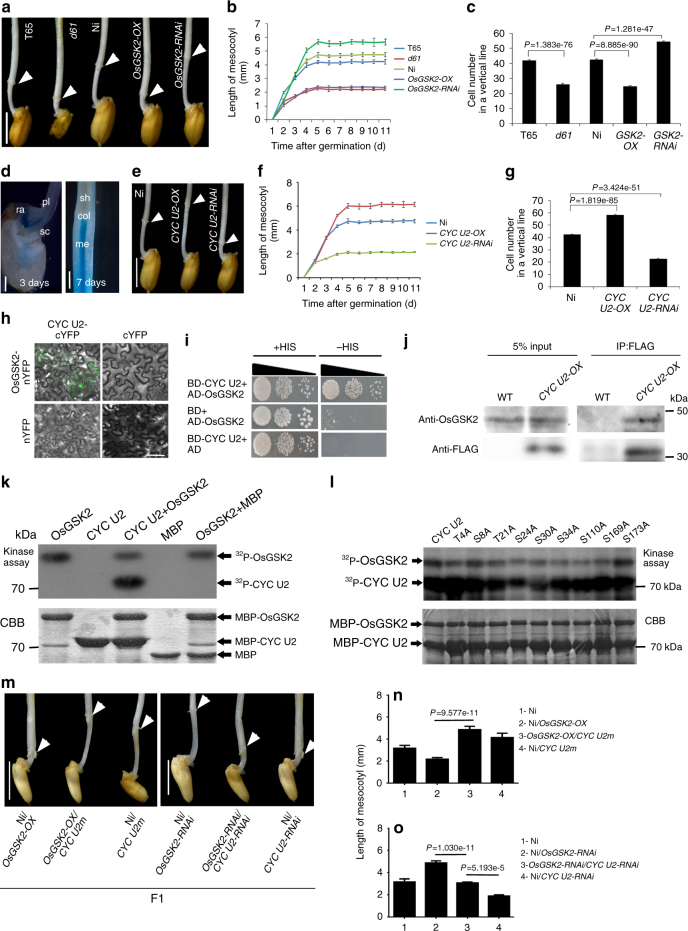
Brassinosteroids regulate mesocotyl elongation through the phosphorylation of CYC U2 by OsGSK2. **a** Morphology of mesocotyls in the BR-related lines on the fifth day after germination. T65 and Ni are wild-type control for *d61* and transgenic lines, respectively. Arrows indicate the coleoptilar nodes. Scale bar, 0.5 cm. **b** Time course of mesocotyl elongation in the BR-related lines in **a**. The sample number is T65 (*n* = 40), *d61* (*n* = 40), Ni (*n* = 43), *OsGSK2-OX* (*n* = 43), *OsGSK2-RNAi* (*n* = 45). **c** Cell number in a vertical line of intact mesocotyls in **a** (*n* = 80). **d** The expression pattern of *CYC U2* in the mesocotyl, as indicated by GUS expression in the *pCYC U2::GUS* transgenic rice. Days after seed soaking are indicated. pl plantule, ra radicle, sc scutellum, col coleoptilar node, me mesocotyl, sh shoot; the arrow indicates the joint region among the plantule, radicle, and scutellum. Scale bar, 1 mm, 0.5 mm. **e** Mesocotyl morphology in *CYC U2* transgenic lines on the fifth day after germination. Arrows indicate the coleoptilar nodes. Scale bar, 0.5 cm. **f** Time course of mesocotyl growth in the plants in **e**. The sample number is Ni (*n* = 43), *CYC U2-OX* (*n* = 40), and CYC U2-RNAi (*n* = 48). **g** Cell number in a vertical line of intact mesocotyls in **e** (*n* = 70). **h**, **i** Interaction between CYC U2 and OsGSK2 in BiFC assays (**h**) and yeast two-hybrid assays (**i**). Scale bar, 100 µm. **j** Interaction between CYC U2 and OsGSK2 in the Co-IP assays. **k** OsGSK2 phosphorylates CYC U2 in vitro. **l** The phosphorylation of the various mutant proteins of CYC U2 by OsGSK2 in vitro. **k**, **l** Upper panel shows autoradiography. Bottom panel shows Coommassie blue staining. **m** The mesocotyl phenotype of the indicated lines for genetic analysis between *CYC U2* and *OsGSK2*. Scale bar, 0.5 cm. **n**–**o** Quantification of the mesocotyls length shown in **m**. In **n**, the samples number of Ni, *Ni/OsGSK2-OX*, *OsGSK2-OX /CYC U2m*, and *Ni/CYC U2m* are 45, 45, 50, and 41, respectively. In **o**, the sample number of Ni, *Ni/OsGSK2-RNAi*, *OsGSK2-RNAi/CYC U2-RNAi*, and *Ni/CYC U2-RNAi* are 45, 40, 45, and 40, respectively. Error bars are SE. *P* value is determined by Welch’s *t* test with Bonferroni correction

Because OsGSK2 is a key negative component in BR signaling^19,20^, we detect whether the different alleles of *OsGSK2* alter the BR signaling. We measured the expression levels of the BR biosynthetic genes, including *D2* and *D11*, which has been reported to be negatively regulated by BR signaling^22,28,29^, in the rice germplasms and transgenic lines with the different *OsGSK2* alleles using RT-qPCR. We found that these genes were up-regulated in the rice germplasms and transgenic lines with the *OsGSK2*^*typeA*^ as compared to that with *OsGSK2*^*typeB*^ (Fig. 2f, g), indicating that the BR signaling is reduced in the rice germplasms containing *OsGSK2*^*typeA*^, a haplotype with high kinase activity. BRs play key roles in regulating plant development, including hypocotyl length in *Arabidopsis*^18^, so we investigated whether OsGSK2-mediated BR signaling regulates rice mesocotyl elongation. Compared to the wild type, the mesocotyls of the BR perception mutant *d61-1* and the *OsGSK2-OX* transgenic plants (with blocked BR signaling) were shorter, whereas those of the *OsGSK2-RNAi* lines (with enhanced BR signaling) were longer on the fifth day after germination (Fig. 3a). To determine the mesocotyl elongation rate of these BR-related lines, we measured the mesocotyl length at different time points. Compared to the wild type, the mesocotyl elongation rate was much higher in the *OsGSK2-RNAi*, but significantly lower in *d61-1* and *OsGSK2-OX*; and in all lines, mecocotyls reached their maximum length on the fifth day after germination (Fig. 3b). To detect whether the BR-promoted mesocotyl elongation is caused by cell division or cell elongation, we longitudinally divided the mesocotyl into three regions from the basal part of the seminal root to the coleoptilar node, named the low, middle, and up regions, respectively, and analyzed the cell size and cell number in each region. Due to abundant aerenchyma formation in the inner layers, we measured the cell number and length in tissue two to three layers away from the epidermis (Supplementary Fig. 3). Analysis of longitudinal sections of the mesocotyl indicated that cells in the low region were the longest, whereas those in the up region were the shortest (Supplementary Fig. 4a, b). We then counted the cell number along the intact mesocotyls and found that *d61-1* and the *OsGSK2-OX* plants had fewer cells in mesocotyl than the wild type, and the *OsGSK2-RNAi* line had more cells than other genotypes, including *OsGSK2-OX* and *Nipponbare* (Ni) (Fig. 3c). Although the *OsGSK2-RNAi* lines (with longer mesocotyls) had longer cells and the *d61-1* and *OsGSK2-OX* (with shorter mesocotyls) had shorter cells than wild type in all three regions (Supplementary Fig. 4a, b), the contribution of cell number to mesocotyl length was 4.62-fold, 2.25-fold, and 2.75-fold greater than that of cell length in these plants, respectively. These results demonstrate that BR signaling promotes rice mesocotyl elongation, primarily by enhancing cell division.

In an early study we found that a member of plant-specific U-type cyclins, *CYC U2*, involved in cell cycle with unknown development process^30^. To identify the function of *CYC U2*, we generated transgenic rice expressing the *GUS* reporter gene driven by the *CYC U2* promoter. Interestingly, *CYC U2* was highly expressed in the joint region among the plantule, radicle, and scutellum, where the monocot mesocotyl will be formed, and subsequently was detected to accompany mesocotyl growth in the vascular bundle (Fig. 3d), suggesting that *CYC U2* may be involved in regulating rice mesocotyl growth. To demonstrate this hypothesis, we detected and found that the mesocotyl of the *CYC U2-OX* line is longer, and the mesocotyl of the *CYC U2-RNAi* is shorter than that of the wild-type Ni on the fifth day after germination (Fig. 3e). Furthermore, the mesocotyl elongation rate was much higher in the *CYC U2-OX* plants, but much lower in the *CYC U2-RNAi* plants than that of Ni (Fig. 3f), and then we counted the cell number along the intact mesocotyls and found that the *CYC U2-OX* plants produced more cells, whereas the *CYC U2-RNAi* plants produced fewer cells than Ni (Fig. 3g). Similar to the effect of BRs on mesocotyl elongation, the contribution of cell number to mesocotyl length was 2.20-fold and 2.70-fold greater than that of cell length in the *CYC U2-OX* and *CYC U2-RNAi* plants, respectively (Supplementary Fig. 4c, d). Therefore, these results suggest that overexpressing *CYC U2* strongly promotes rice mesocotyl elongation primarily by enhancing cell division, and indicate that BRs might promote mesocotyl cell division through *CYC U2*.

In mammals, the stability and activity of many cyclins depend on their GSK3β-regulated phosphorylation^31–33^. Therefore, we investigated whether OsGSK2 and CYC U2 can interact with each other, and found that they can interact in BiFC, yeast two-hybrid assays, and co-immunoprecipitation (Co-IP) in vivo (Fig. 3h–j). We then conducted in vitro kinase assays and found that OsGSK2 can strongly phosphorylate CYC U2 in vitro (Fig. 3k). To test whether OsGSK2 can phosphorylate CYC U2 in planta, we firstly immunoprecipitated the CYC U2-FLAG protein with anti-FLAG beads from the *CYC U2-OX* mesocotyls, which was then treated with calf intestinal alkaline phosphatase (CIP). To avoid protein degradation, we added MG132 and a cocktail of proteinase inhibitors in the protein extracts. We detected two bands of CYC U2-FLAG with anti-FLAG antibody, and CIP treatment can induce the reduction of the upper band, indicating it is corresponding to the phosphorylated CYC U2-FLAG (CYC U2-FLAG-P) (Supplementary Fig. 5a). Second, we compared the ratio of the phosphorylated to the unphosphorylated CYC U2-FLAG for the IPed CYC U2-FLAG from the *CYC U2-OX* mesocotyls grown on medium without or with castasterone (CS, the most active BRs in rice^34^) or bikinin (a well-known OsGSK2 inhibitor). We found that CS and bikinin treatment can significantly reduce the level of the CYC U2-FLAG-P (Supplementary Fig. 5b). Third, we compared the phosphorylation level of the CYC U2-FLAG in the F1 plants of Ni and *CYC U2-OX* or *OsGSK2-OX* and *CYC U2-OX*, and found that overexpression of OsGSK2 can strongly enhance the phosphorylation of CYC U2-FLAG (Supplementary Fig. 5c). Finally, we identified its putative phosphorylation sites by OsGSK2, and then mutated the putative phosphorylation sites of CYC U2 to Ala and constructed the mutant proteins T4A, S8A, T21A, S24A, S30A, S34A, S110A, S169A, and S173A. Kinase assays showed that the phosphorylation levels of T21A, S24A, and S30A were significantly reduced (Fig. 3l), indicating that these sites are likely the major sites being phosphorylated by OsGSK2 kinase. We then generated transgenic rice expressing *CYC U2m* (we mutated the OsGSK2-phosphorylated sites in CYC U2 to Ala and defined the *CYC U2*^*T21AS24AS30A*^ as *CYC U2m*), and found that the mesocotyls of the *CYC U2m* lines were much longer than that of *CYC U2-OX*, when the expression level of *CYC U2* and *CYC U2m* is similar between the two lines of each pair (Supplementary Fig. 5d, e). These results indicated that BRs regulate CYC U2 phosphorylation through OsGSK2 in planta, and the OsGSK2-phosphorylated modification on CYC U2 play a vital role in mesocotyl elongation.

To explore whether OsGSK2-phosphorylated CYC U2 genetically participates in the BR-controlled mesocotyl elongation, we firstly detected the BR sensitivity of mesocotyl elongation regulated by *CYC U2*. We planted the *CYC U2-OX*, *CYC U2-RNAi*, and *CYC U2m* transgenic rice on the medium containing the different concentrations of CS to measure the mesocotyl elongation, and found that *CYC U2-RNAi* transgenic rice was the most insensitive to BRs in mesocotyl elongation (Supplementary Fig. 4e). Second, we measured the sensitivity of the *CYC U2-OX* and *CYC U2m* transgenic rice on mesocotyl elongation inhibition to different concentrations of brassinozole (BRZ), a BR biosynthesis inhibitor, and we found that the *CYC U2m* line was highly insensitive to BRZ as compared to the *CYC U2-OX* line and the wild type (Supplementary Fig. 4f). Third, we crossed the *OsGSK2-OX* plants with plants expressing *CYC U2m* and found that the mesocotyl length of the *OsGSK2-OX/CYC U2m* hybrid lines was similar to that of *CYC U2m* plants (Fig. 3m, n), suggesting that *CYC U2m* suppressed the mesocotyl phenotype of *OsGSK2-OX*. In addition, the mesocotyls of the *OsGSK2-RNAi*/*CYC U2-RNAi* hybrid plants were shorter than that of the *OsGSK2-RNAi* (Fig. 3m, o), indicating that *CYC U2-RNAi* suppresses the mesocotyl phenotype of the *OsGSK2-RNAi* line. Furthermore, we found that *CYC U2-RNAi* and *CYC U2m* still slightly responded to BR and BRZ with significantly reduced sensitivity, and the mesocotyls of the *OsGSK2-RNAi*/*CYC U2-RNAi* hybrid plants were longer than that of *CYC U2-RNAi*. It suggests that there may be other components downstream of BR signaling and *OsGSK2*, which are additive to *CYC U2* in mesocotyl elongation. Therefore, these results suggest that *CYC U2* acts downstream of *OsGSK2* to regulate BR-related mesocotyl elongation.

### SLs/D3 degrade the OsGSK2-phosphorylated CYC U2

**Fig. 4:**
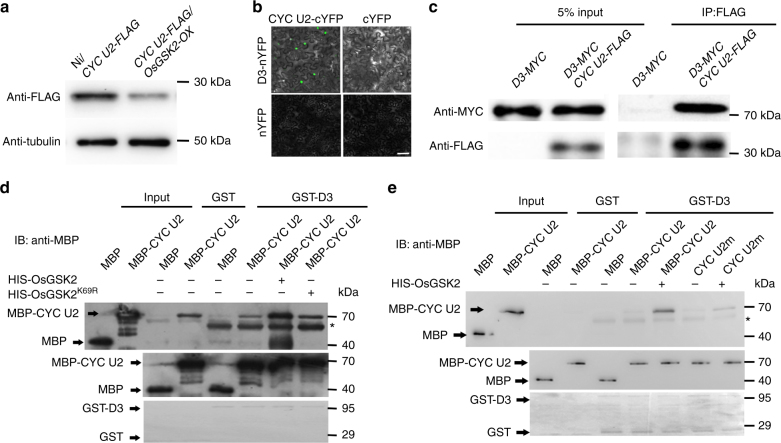
The OsGSK2-phosphorylated CYC U2 interacts with D3. **a** CYC U2 protein levels in the Ni/*CYC U2-FLAG* and *CYC U2-FLAG/OsGSK2-OX* F_1_ hybrids lines. The wild-type *Nipponbare* crossed with *CYC U2-FLAG* as a control. The mesocotyls are pre-treated with cycloheximide (CHX) for 3 h. Anti-FLAG was used to detect CYC U2-FLAG fusion protein level, and anti-tubulin was used for equal loading. **b** Interactions between CYC U2 and D3 in BiFC assays. Scale bar, 100 µm. **c** Interaction between CYC U2 and D3 in the Co-IP assays. The proteins were expressed and extracted from tobacco leaf and immunoprecipitated by anti-FLAG M2 magnetic beads. Gel blots were probed with anti-FLAG or anti-MYC antibody. **d** D3 interacts with the OsGSK2-phosphorylated CYC U2 in GST pull-down assays. The kinase dead type, OsGSK2^K69R^-HIS, is as the negative control to OsGSK2-HIS. MBP-CYC U2 was preincubated with OsGSK2-HIS and OsGSK2^K69R^-HIS, respectively, and then subjected to the pull-down assay. The top panel is the result of pull-down assay immunoblot with anti-MBP; the bottom and middle panels are the protein loading control. Asterisk indicates the nonspecific binds. **e** The CYC U2m exhibits a reduced interaction with D3 in GST pull-down assay. Asterisk indicates the nonspecific binds

However, how OsGSK2-phosphorylated CYC U2 causes the reduced cell division in mesocotyl is still unknown. It was reported that phosphorylation in the N or C terminus of some cyclins by GSK3β in mammals is related to their stability^31,35^. We found that the phosphorylated sites in CYC U2 (T^21^S^24^S^30^) are located in the N terminus, so we detected whether the stability of CYC U2 is regulated by OsGSK2. Interestingly, we found that the protein level of CYC U2 was significantly reduced in the *OsGSK2-OX* background compared to that in the wild-type Ni background (Fig. 4a), indicating that OsGSK2 promotes CYC U2 degradation. A previous study showed that SLs inhibit mesocotyl elongation by decreasing cell division via an unknown mechanism, which depends on SL receptor D14 and the interactor D3 (ref. 9,36,). Because D3 is an F-box-type E3 ligase^14–16^, we speculated that D3 might be involved in degradation of CYC U2. Interestingly, although we detected a physical interaction between CYC U2 and D3 in BiFC analysis and Co-IP in vivo (Fig. 4b, c), we did not detect their interaction in a yeast two-hybrid assay (Supplementary Fig. 6a). In addition, we performed in vitro GST pull-down assay and found that the amount of CYC U2 detected by the GST-D3 pull-down assay was similar to that detected by the negative control (Supplementary Fig. 6b), indicating that the interaction between CYC U2 and D3 was not detected by in vitro GST pull-down assay. Therefore, we hypothesized that certain modifications of CYC U2 in planta might be required for their interaction.

**Fig. 5:**
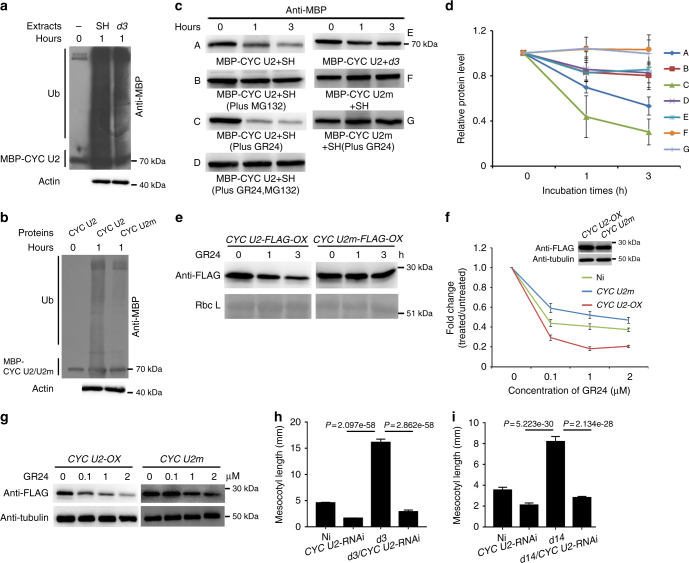
SL-inhibited mesocotyl elongation depends on the degradation of the OsGSK2-phosphorylated CYC U2 by D3. **a** The ubiquitination assay of CYC U2 in SH and *d3* mutant. Endogenous actin detected with actin antibody showed the equal amounts of crude extracts from SH and *d3* plants incubated. SH is the background of *d3*. **b** The ubiquitination assay of CYC U2 and CYC U2m in SH. **c** The cell-free degradation assays for detecting degradation of CYC U2 and CYC U2m in SH and *d3* with or without GR24 treatment. The equal recombinant proteins were incubated with the equal plant crude extracts. **d** Quantification analysis for **c**. The relative levels of MBP-CYC U2 and MBP-CYC U2m incubated with the indicated plant extracts at 0 h were defined as “1.” The degradation assay has been independently repeated for three times (means ± SD). **e** The protein levels of CYC U2-FLAG and CYC U2m-FLAG in the corresponding transgenic rice incubated with 5 µM GR24 at the indicated time points. Rbc L was used as a loading control. **f** The sensitivity assays of *CYC U2* and *CYC U2m* transgenic rice responding to SL treatments in mesocotyl length. The close-up view indicates the transgenic rice used in (**f**, **g** with the similar protein level of the transgenic gene without GR24 treatment. Error bars are SE (*n* = 15). Ni is the wild-type control. **g** CYC U2-FLAG or CYC U2m-FLAG protein levels in the mesocotyls of *CYC U2-OX* or *CYC U2m* transgenic lines from **f**. Endogenous tubulin detected with tubulin antibody showed equal loading. **h** The mesocotyl length of the indicated lines for genetic analysis between *CYC U2* and *D3*. Ni is the wild-type control. Data are mean ± SE. The sample number of Ni, *CYC U2-RNAi*, *d3*, and *d3/CYC U2-RNAi* are 44, 32, 31, and 50, respectively. **i** The mesocotyl length of the indicated lines for genetic analysis between *CYC U2* and *D14*. Ni is the wild-type control. Data are mean ± SE (*n* = 35). **h**, **i** *P* values were determined by Welch’s *t* test with Bonferroni correction

To explore whether the interaction between D3 and CYC U2 is dependent on the phosphorylated modification on CYC U2 by OsGSK2, we performed the modified in vitro pull-down assay, and found that the amount of the unphosphorylated CYC U2 or the CYC U2 preincubated with OsGSK2^K69R^ detected by the GST-D3 pull-down assay were less than the amount of the OsGSK2-phosphorylated CYC U2, and were similar to that detected by the negative control (Fig. 4d), supporting that the OsGSK2-phosphorylated CYC U2 interacts more strongly with D3 than the unphosphorylated CYC U2. Furthermore, even in the presence of OsGSK2, the interaction between D3 and CYC U2m was strongly reduced compared to the interaction between D3 and CYC U2 (Fig. 4e). These results indicate that the phosphorylated modification of CYC U2 by OsGSK2 is essential for its interaction with D3. To investigate whether D3 directly regulates CYC U2 ubiquitination and degradation, we conducted a CYC U2 ubiquitination assay and found that CYC U2 was more strongly ubiquitinated by protein extract from the Shiokari (SH) seedlings (the wild-type background of *d3 mutant*) than by protein extract from *d3* seedlings (Fig. 5a), indicating that D3 is required for the ubiquitination of CYC U2 in vivo. Furthermore, the ubiquitination level of CYC U2m was apparently lower than that of the wild-type CYC U2 in SH (Fig. 5b), suggesting that ubiquitination of CYC U2 by D3 is dependent on its phosphorylation by OsGSK2. We then investigated whether the SL/D3-induced ubiquitination results in CYC U2 degradation using a cell-free protein degradation system^17,37^. We incubated the recombinant protein MBP-CYC U2 (pre-phosphorylated by OsGSK2) with protein extracts from SH or *d3* plants and monitored the amount of MBP-CYC U2 remaining in the reactions by immunoblot analysis at the indicated time points. The CYC U2 degradation rate was much lower in *d3* than that in SH protein extracts and was enhanced by protein extracts from SH treated with GR24 (a synthetic analog of SLs). Furthermore, CYC U2m was more stable than CYC U2 in protein extracts from SH seedlings both with and without GR24 treatment (Fig. 5c, d), and MG132 treatment increased the stability of CYC U2 in protein extracts from SH plants both with and without GR24 treatment (Fig. 5c, d). In addition, we detected the effect of GR24 on CYC U2 stability in *d14* and *d3* mutants, and found that CYC U2 was stable in protein extracts from *d14* and *d3* seedlings both with and without GR24 treatment compared to that in SH (the wild-type) seedlings (Supplementary Fig. 7), suggesting that the CYC U2 stability is decreased by SLs via D14 and D3. To further detect whether SLs regulate CYC U2 stability in planta, we measured the CYC U2 and CYC U2m protein levels in the *CYC U2-OX* and *CYC U2m* transgenic plants treated by 5 µM GR24 at the indicated time points, and found that CYC U2m was more stable than CYC U2 after GR24 treatment in vivo (Fig. 5e). Taken together, these results indicate that SLs/D3-induced degradation of CYC U2 depends on the phosphorylated modification on CYC U2 by OsGSK2.

To investigate whether the degradation of OsGSK2-phosphorylated CYC U2 is required for SL-inhibited mesocotyl elongation in vivo, we performed a series of genetic analyses. We firstly planted the *CYC U2-OX* and *CYC U2m* transgenic rice in the medium containing the gradient concentrations of GR24 to assess the ability of mesocotyl elongation. We selected transgenic lines with similar transgenic protein levels of CYC U2 and CYC U2m (the close-up view in Fig. 5f), which have the similar mesocotyl length without GR24 treatment (Supplementary Fig. 8), for this assessment; and we found that the *CYC U2m* transgenic line was less sensitive to the GR24-induced inhibition of mescotyl growth than the *CYC U2-OX* line (Fig. 5f), which coincides with the CYC U2s levels in vivo that the CYC U2m protein was more stable than the CYC U2 under the gradient GR24 treatment in the mesocotyl (Fig. 5g). Second, we knocked down *CYC U2* expression in the *d3* and *d14* mutants, and found that the mesocotyl length of the *d3/CYC U2-RNAi* and the *d14/CYC U2-RNAi* plants was all similar to that of the *CYC U2-RNAi* plants (Fig. 5h, i), suggesting that knockdown of *CYC U2* suppresses mesocotyl elongation in *d3* and *d14*. These genetic analyses demonstrated that the SL-regulated mesocotyl elongation relies on the *CYC U2*. Therefore, we conclude that the OsGSK2-induced phosphorylation of CYC U2 is required for its interaction with and degradation by D3 in the SL-controlled mesocotyl elongation in rice.

## Discussion

Although mesocotyl length is a crucial developmental trait for many cereal crops in biology and agriculture, and imminently required to improve the crop adapting to modern cultivation modes, the genetic regulatory factors and molecular mechanisms of mesocotyl elongation and domestication have not been uncovered. Here, we identified that OsGSK2-determined degradation of CYC U2 by D3 participates in the mesocotyl elongation and domestication via integrating the SL and BR signaling to specifically regulate mesocotyl cell division. Thus, our results demonstrated a mechanism that two distinct signaling pathways interdependently regulate a cell cycle-specific cyclin’s stability to integrate developmental processes in rice, which could be used in other systems. In addition, the natural selection of *OsGSK2* plays a key role in rice mesocotyl variation and hormone signaling domestication.

In this study, we demonstrate that a conserved GSK3 kinase OsGSK2 determines the natural variation in mesocotyl elongation and the directional selection in mesocotyl domestication. It was previously reported that mesocotyl of upland rice is longer than that of lowland rice, which facilitates the upland rice seedling emergence from deep soil^38–40^. Thus, the mesocotyl length is an important trait for rice to adapt to different environments and human cultivation habits during rice domestication and cultivation. Our GWAS, genetic and biochemical analyses, and allelic gene frequency analysis revealed that the two major natural alleles of *OsGSK2* (*OsGSK2*^*typeA*^, with high kinase activity, and *OsGSK2*^*typeB*^/*OsGSK2*^*typeB*^*-like*, with weak kinase activity) control mesocotyl variation during rice domestication. Based on the four SNPs in coding region, we found that the diversity of *OsGSK2* haplotypes in the 947 cultivated rice population was significantly reduced compared to that in the 397 *O. rufipogon* population (Supplementary Fig. 2c, d), suggesting that this locus likely belongs to a selective sweep in crop domestication^41^. We calculated the *F*_ST_ between *O. sativa* and *O. rufipogon* genome-wide, and found that the *F*_ST_ level in the *OsGSK2* locus is significantly higher than the average *F*_ST_ level in the whole genome (Fig. 2e), ranking at the 95.5th percentile among all genes in the whole genome, indicating that the *OsGSK*2 locus participates in the domestication from wild rice to cultivated rice. This is further demonstrated by the ratio of allelic gene frequency between *OsGSK2*^*typeA*^ and *OsGSK2*^*typeB/typeB*^*-like*, nearly 1:9 in *O. rufipogon* population but 3:1 in the cultivated rice population, including *japonica* population and *indica* population (Supplementary Fig. 2c, d). In addition, the ratio of allelic gene frequency between *OsGSK2*^*typeA*^ and *OsGSK2*^*typeB*^ in *japonica* population was similar to that in *indica* population (among 947 cultivated rice population), which is nearly 3:1, suggesting that the natural/artificial selection directionally and non-randomly favors the *OsGSK2*^*typeA*^ allele over *OsGSK2*^*typeB*^ allele whenever in the domestication from wild rice to *japonica* or to *indica*. Furthermore, within its LD region, the *OsGSK2* locus has the highest *F*_ST_ level (Fig. 2e), and there is no other linked genes with obvious functional evidences identified by the domestication sweeps^26^. Moreover, the different alleles of *OsGSK2* have been demonstrated to control the rice mesocotyl variation (Fig. 2a–d). Taken together, it was concluded that the different alleles of *OsGSK2* was undergone directional selection during the domestication from the *O. rufipogon* to the cultivated rice.

Our discovery of the natural variation of *OsGSK2*, a key component in the BR signaling pathway, serves as an excellent example for exploring the natural variation in other BR-related traits associated with *GSK3s*. Plant GSK3s regulate diverse developmental programs by phosphorylating different substrates to increase plant adaption to the environment, including stomatal formation, root hair initiation, lateral root development, and leaf erectness^30,42,43^. During the domestication from the wild species to the cultivated rice, the coordinated alterations in multiple phenotypes are required to allow the plants to adapt to diverse environments and human habits. Therefore, most likely, *OsGSK2* is also involved in regulating the domestication of other traits besides mesocotyl in rice. Especially, BRs have been reported to significantly regulate the leaf angle in rice^30,44^. In addition, the previous studies have reported that *OsGSK2-OX* transgenic rice exhibits the erect leaf with the reduced leaf angle, and the *OsGSK2-RNAi* lines exhibit the enlarged leaf angle^29,30^. In this study, we also found that the transgenic rice with *OsGSK2*^*typeA*^ (with high kinase activity) exhibited the reduced leaf angle compared to the *OsGSK2*^*typeB*^ (with weak kinase activity) transgenic rice (Supplementary Fig. 9). Therefore, the domestication of *OsGSK2* might be also important for regulating other traits in rice.

Furthermore, although BES1 has been reported to be a common component shared by SL and BR signaling in *Arabidopsis* to regulate signal-specific development^17^, how these two signals crosstalk to regulate plant development is largely unknown. SLs were first identified as compounds that promote parasitic plant germination^45^. Treatment with exogenous GR24 stimulates plant germination^46^, but inhibits hypocotyl and mesocotyl elongation in *Arabidopsis* and rice, respectively^9,47^. While BRs play a key role in promoting hypocotyl elongation in *Arabidopsis*^18^ and mesocotyl elongation in rice demonstrated by our study. However, successful seedling establishment requires both germination and hypocotyl/mesocotyl elongation for plants to outgrow of the soil. Therefore, rice may employ BR signaling to balance the contradictory effect of SLs on germination and hypocotyl/mesocotyl elongation, which can be achieved by the degradation of the OsGSK2-phosphorylated CYC U2 via D3.

**Fig. 6:**
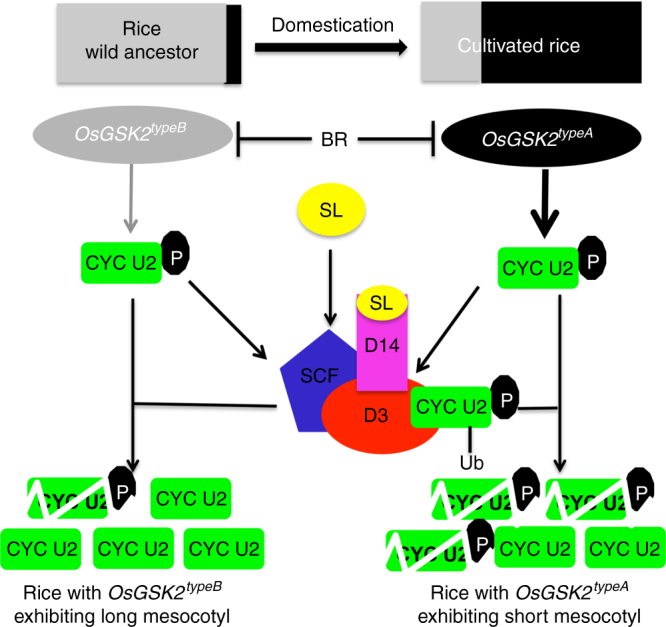
A proposed working model for mesocotyl elongation and domestication. Gray indicates *OsGSK2*^*typeB*^ with low kinase activity, and black indicates *OsGSK2*^*typeA*^ with high kinase activity

In conclusion, we propose a model explaining how the degradation of the OsGSK2-phosphorylated CYC U2 by D3 regulates mesocotyl elongation and domestication in rice (Fig. 6). The U-type cyclin CYC U2 regulates rice mesocotyl elongation via cell division. SLs inhibit rice mesocotyl elongation via the degradation of CYC U2 by D3, which is dependent on the phosphorylation of CYC U2 by OsGSK2, whose kinase activity is inhibited by BR signaling. Specific natural alleles of *OsGSK2* with different kinase activities have been selected during rice domestication, and are responsible for natural variation in mesocotyl elongation. Our findings reveal an important mechanism for mesocotyl elongation and domestication, and the natural alleles of *OsGSK2* provide promising resources for molecular design in rice improvement.

## Methods

### Plant materials

The rice cultivar Ni, as well as *CYC U2-OX*, *CYC U2-RNAi*, *CYC U2m*, *pCYC U2::GUS*, *GSK2-OX*, and *GSK2-RNAi* lines in the Ni background, *OsGSK2*^*TypeA*^*-OX* and *OsGSK2*^*TypeB*^*-OX* lines (Kasalath background), the mutants *d61-1* (cv. Taichung 65, T65 background), *d3* (SH and Ni background), and *d14* (SH background), hybrid lines *Ni*/*OsGSK2-OX*, *Ni*/*CYC U2m*, *OsGSK2-OX*/*CYC U2m*, *Ni*/*OsGSK2-RNAi*, *Ni*/*CYC U2-RNAi*, *OsGSK2-RNAi*/*CYC U2-RNAi*, and *d3* (Ni background)/*CYC U2-RNAi*, and CSSLs lines which were constructed with African cultivated rice CG14 (*O. glaberrima*), as the donor parent and the *japonica* variety WYJ as the recurrent parent^48^, were used in this study. To measure mesocotyl length, rice plants, after germination, were grown in water in darkness at 28 °C in climate chambers for the indicated number of days. We detected protein levels in the transgenic plants by anti-FLAG (M20008; Abmart, 3000-fold dilution), and anti-Tubulin as the control (AT819; Beyotime, 2000-fold dilution).

### Vector construction and plant transformation

To generate *CYC U2m* plants expressing *CYC U2*^*T21AS24AS30A*^, the ORF of the mutated *CYC U2* was cloned in-frame with FLAG into the pCambia1306 vector and driven by the *CYC U2* promoter (2 kb upstream of the start codon), and the plants expressing *OsGSK2*^*typeA*^*-OX* or *OsGSK2*^*typeB*^*-OX* were generated by cloning the indicated ORFs in-frame with FLAG into the pCambia1306 vector and driven by 35S promoter. To produce the *RNA* interference lines, the portions of *CYC U2* cDNA (−168 to 199) was amplified and inserted into pTCK303 (ref. 49) driven by 35S promoter. In addition, the plants expressing *CYC U2-OX*, *pCYC U2::GUS*, *OsGSK2-OX*, and *OsGSK2-RNAi* were constructed in our previous studies^29,30^. All the constructs were introduced into *Agrobacterium tumefaciens* strain EHA105 and transformed into the indicated background.

### Yeast two-hybrid assays

The full-length coding sequences of *CYC U2* and *OsGSK2* were cloned into the pAD502 vector, and the full-length coding sequences of *D3* and *CYC U2* were cloned into the pDBLeu vector, respectively. These constructs or the corresponding empty vectors were co-transformed into the yeast strain AH109 and grown on SD medium lacking Leu, Trp, and His.

### BiFC analysis

*CYC U2* was cloned into pXY104, and *OsGSK2* and *D3* were cloned into pXY106, respectively. The transformed *Agrobacterium* were infiltrated into young leaves of *Nicotiana benthamiana*. The expression of YFP fluorescent proteins was observed by confocal microscopy (Zeiss).

### Pull-down assays

To investigate the interaction between CYC U2 and D3 in vitro, the ORF of D3 was amplified and cloned into pGEX4T-1, and the CYC U2s (including CYC U2 and CYC U2^T21AS24AS30A^) were cloned into pMAL-c2x, respectively. The recombinant fusion proteins were expressed in *Escherichia coli* and purified with the corresponding affinity beads. The pull-down assays were performed by incubating GST or D3-GST-coupled beads with MBP-CYC U2s proteins for 3 h at 4 °C, washed thoroughly, separated by 12% sodium dodecyl sulfate polyacrylamide gel electrophoresis (SDS-PAGE), and detected by immunoblot analysis with anti-MBP antibody (prepared by our laboratory, 3000-fold dilution).

To determine whether the interaction between CYC U2 and D3 is dependent on the phosphorylation of CYC U2 by OsGSK2, OsGSK2-HIS and OsGSK2^K69R^-HIS fusion proteins were constructed. Prior to the incubation of MBP-CYC U2s and D3-GST in the pull-down assays, the MBP-CYC U2s were incubated with OsGSK2-HIS and OsGSK2^K69R^-HIS, respectively, for 1 h at 37 °C in kinase assay buffer (50 mM Tris-HCl, pH 7.4, 10 mM MgCl_2_, 1 mM dithiothreitol (DTT), 100 μM ATP), and then subjected to the pull-down assay. Pull-down-related blots were shown in Supplementary Fig. 11.

### Co-immunoprecipitation

To detect that CYC U2 can interact with OsGSK2, the rice were ground to a fine powder in liquid nitrogen and solubilized with 2× extraction buffer (100 mM Tris-HCl, pH7.5, 300 mM NaCl, 2 mM EDTA, pH 8.0, 1% Triton X-100, 10% glycerol, and a protease inhibitor cocktail). The extracts were centrifuged at 20,000 × *g* for 10 min, and the resultant supernatant was incubated with prewashed anti-FLAG M2 beads (Sigma-Aldrich) for 3 h at 4 °C, and then the beads were washed six times with the 2× extraction buffer. The immunoprecipitates were eluted with 1× SDS sample buffer, separated on a 12% SDS-PAGE gel, transferred to nitrocellulose membrane (Amersham Biosciences), and detected with corresponding antibodies. Anti-GSK2 (AbP80050-A-SE, 2000-fold dilution) was purchased from Beijing Protein Innovation.

To detect that CYC U2 can interact with D3, the full-length coding sequence of CYC U2 was cloned into pCambia 1306 vector which fuse with FLAG tag and D3 was cloned into the pCambia 1300 vector which fuse with MYC tag, and then transformed into the *Agrobacterium* strain GV3101 and coinjected into young leaves of *N*. *benthamiana*. The protein extraction referred to the above method. The anti-FLAG (M20008; Abmart, 3000-fold dilution) and anti-MYC antibody (M20002; Abmart, 3000-fold dilution) was used to test the results. Co-IP-related blots were shown in Supplementary Figs. 10, 11.

### In vitro kinase assay

For the in vitro kinase assay, the fusion proteins were added to the reaction buffer (50 mM Tris-HCl, pH 7.4, 10 mM MgCl_2_, 1 mM DTT, 10 μM ATP, and 1 μL of [γ-^32^P]ATP). The kinase reaction was conducted in a 37 °C water bath for 1 h^50^, followed by separation of the proteins by 12% SDS-PAGE and detected via autoradiography. Kinase assay-related blots were shown in Supplementary Fig. 10.

### Cell-free protein degradation assay

SH and *d3* seedlings were grown at 28 °C for 16 h (day) and 26 °C for 8 h (night), harvested, and ground to a fine powder in liquid nitrogen. Total protein was extracted in degradation buffer with equal amounts of powder (25 mM Tris-HCl, pH 7.5, 10 mM NaCl, 10 mM MgCl_2_, 5 mM DTT, 4 mM phenylmethylsulfonyl fluoride, and 10 mM ATP). Cell debris was removed by 2 × 10 min centrifugations at 17,000 × *g* at 4 °C. Total protein extracts prepared from different seedlings were adjusted to equal concentration with the degradation buffer, in which the equal recombinant proteins were incubated at different times. The equal extracts (100 µL extract containing 500 µg total proteins) were added to the tubes containing equal amounts of recombinant proteins for each assay and incubated at 28 °C for various periods of times^17,29^. Some reactions also contained the 50 μM MG132 and 10 μM GR24 as indicated. The recombinant proteins were pre-phosphorylated by OsGSK2 as indicated for the pull-down assays. The band intensity was quantified using the ChemiScope analysis software. Cell-free protein degradation-related blots were shown in Supplementary Figs. 12, 13.

### In vitro ubiquitination assay

Purified MBP-CYC U2s protein bound to Amylose Resin (NEB) was incubated at 28 °C with equal amounts of crude extracts from SH and *d3* plants in degradation buffer with 50 μM MG132. After incubation for the indicated time period, MBP-CYC U2s fusion proteins were eluted at room temperature. The supernatants were separated by SDS-PAGE gel and detected by immunoblotting with antibody against MBP (prepared by our laboratory, 3000-fold dilution)^17^. The equal amounts of plant crude extracts were detected by anti-Actin (AA128; Beyotime, 2000). Ubiquitination-related blots were shown in Supplementary Fig. 12.

### Phosphorylation assay in planta

To detect CYC U2 phosphorylated band in vivo, the mesocotyls of 5-day *CYC U2-OX* plants and the seedlings of the F1 hybrids lines were used to extract protein and the immunoprecipitated CYC U2-FLAG was used for detection with anti-FLAG (for the method refer to Co-IP part). The 50 μM MG132 was pre-added to the protein extraction buffer. For CIP treatment, the plants were grown in darkness in water, the immunoprecipitated protein was treated with 200 U CIP, or the water only containing the reaction buffer of CIP (as control) at 37 °C for 1 h. For CS or bikinin treatment, the *CYC U2-OX* plants were grown in the medium with dimethyl sulfoxide (DMSO) (as control, because CS and bikinin were dissolved in DMSO), the 100 nM CS or 50 μM bikinin (GSK2 inhibitor), respectively, in darkness for 5 days. Phosphorylation-related blots were shown in Supplementary Fig. 13.

### GWAS analysis and SNP phylogenetic analysis

Approximately 30 seeds per accession from a collection of 510 accessions^51^ were germinated, and at least 15 normal seedlings were subjected to mesocotyl length measurement using ImageJ for GWAS analysis (Supplementary Data 1). In total, we used 4, 192, and 129 SNPs^51^ with a minor allele frequency of >0.05 for association analysis. Association analysis was preformed via FaST-LMM (C++ Version 2.07) software^52,53^. The kinship matrix calculated previously^51^, which could represent the population structure, was used in the LMM as random effect. With the number of SNPs analyzed (*n* = 4, 192, 129), the threshold for significance was estimated to be approximately *P* = 1 × 10^−8^ (i.e., 0.05/4, 192, 129) and suggestive *P* = 1 × 10^−7^ (0.5/4, 192, 129) by the Bonferroni correction method^54,55^.

A list of SNPs in the *GSK2* promoter (upstream 2000 bp) and CDS of the 510 accessions was downloaded from the database RiceVarMap database^56^, and these sequences were aligned using the MEGA 6.0 software. A phylogenetic tree was constructed using the neighbor-joining method in MEGA 6.0.

### Quantification and statistical analysis

The data for qRT-PCR were collected with a Bio-Rad CFX system, and qRT-PCR results were analyzed by the 2^−ΔΔ^CT method using the Bio-Rad CFX Manager 3.1 software. Welch’s two-sample *t* test and Welch’s *t* test with Bonferroni correction for multiple comparisons were used to analyze the significance of differences. The statistical analysis was performed in R software (version 3.4.3) and the *P* values have added in figures.

### Primers sequences

The sequences for all primers used in this study are listed in Supplementary Data 3.

### Data availability

The data supporting the findings of this study are available in this article and its Supplementary Information and Data files, or are available from the corresponding author upon request.

## Electronic supplementary material

Supplementary Information

Descriptions of Additional Supplementary Files

Supplementary Data 1

Supplementary Data 2

Supplementary Data 3
