# Supplementary material for: Natural selection of a GSK3 determines rice mesocotyl domestication by coordinating strigolactone and brassinosteroid signaling
Source: Nat Commun. 2018 Jun 28;9:2523. doi: 10.1038/s41467-018-04952-9 (PMC6023860; doi:10.1038/s41467-018-04952-9)

Natural selection of a *GSK3* determines rice mesocotyl domestication by coordinating strigolactone and brassinosteroid signaling

Sun *et al.*


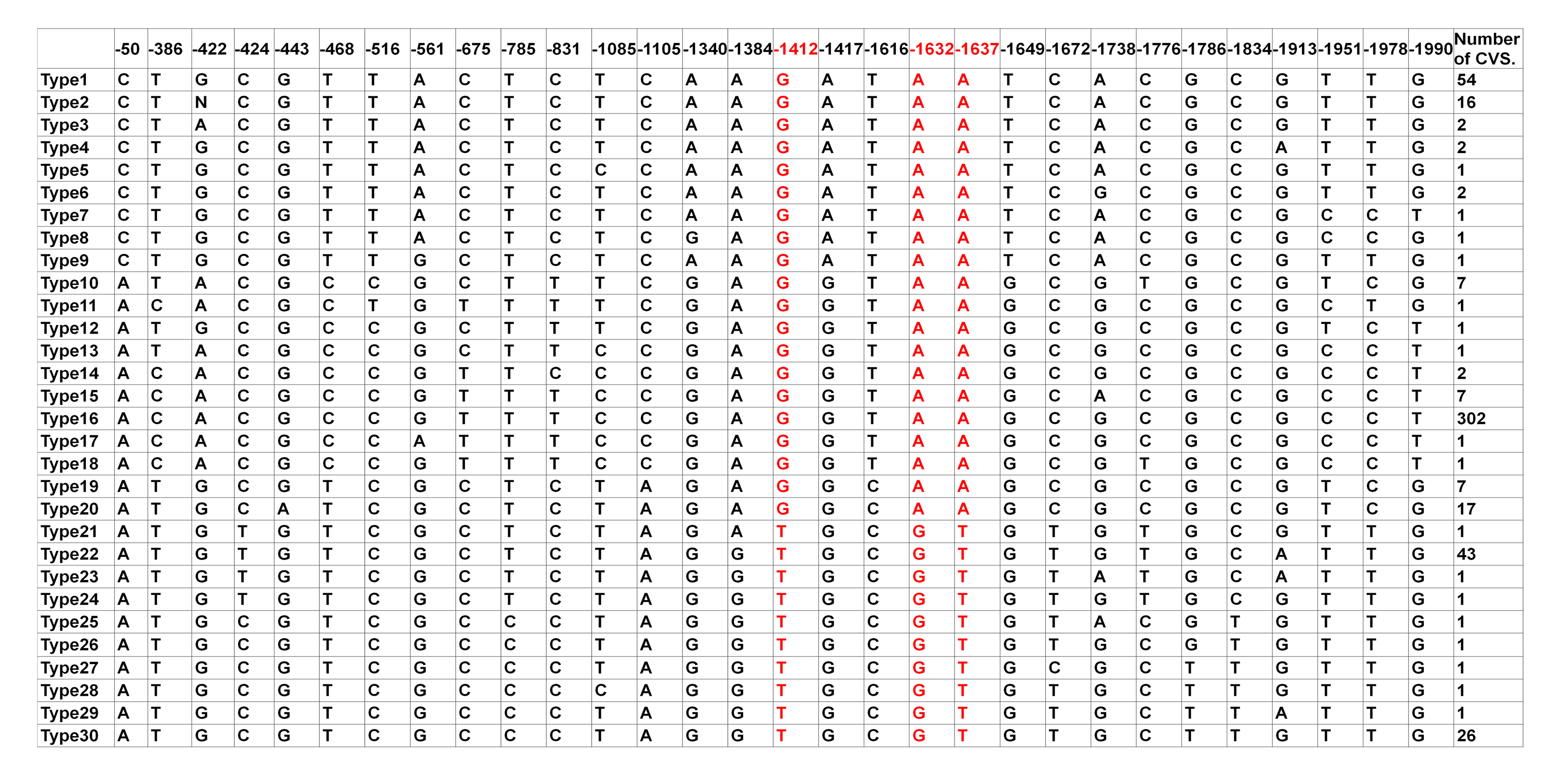


**Supplementary Figure 1. Polymorphic SNPs in the *OsGSK2* promoter regions from the 504 accessions used for GWAS.** Three consensus SNPs (shown in red) are suggested to be responsible for the two classes characterized by phylogenetic analysis.


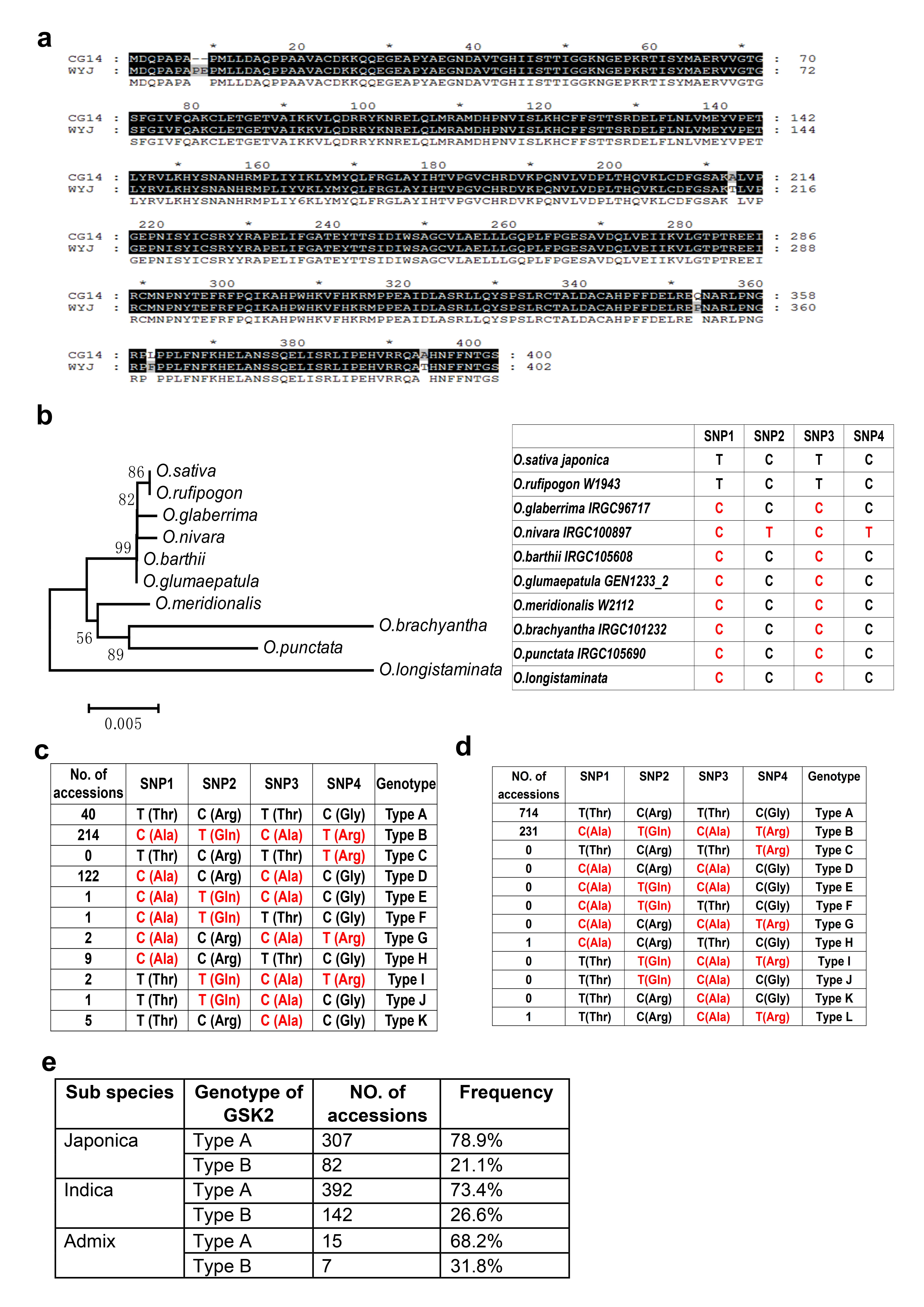


**Supplementary Figure 2. Allele frequency and sequence alignment of *OsGSK2* in the wild species and the cultivated rice. (a)** Protein sequence alignment of OsGSK2 between WYJ and CG14. **(b)** Left: Phylogeny (Neighbor-Joining tree) based on *OsGSK2* cDNA sequences. Bootstrap values (>50%) from 10,000 replicates are given next to the branches. Right: genotypes of *OsGSK2* orthologs in the *Oryza* genus. **(c-d)** Genotypes of *OsGSK2* in *Oryza rufipogon* accessions (c) and cultivated rice (d). **(e)** The allelic gene frequency between *OsGSK2^typeA^* and *OsGSK2^typeB^* in *japonica* population and *indica* population among 947 cultivated rice accessions.


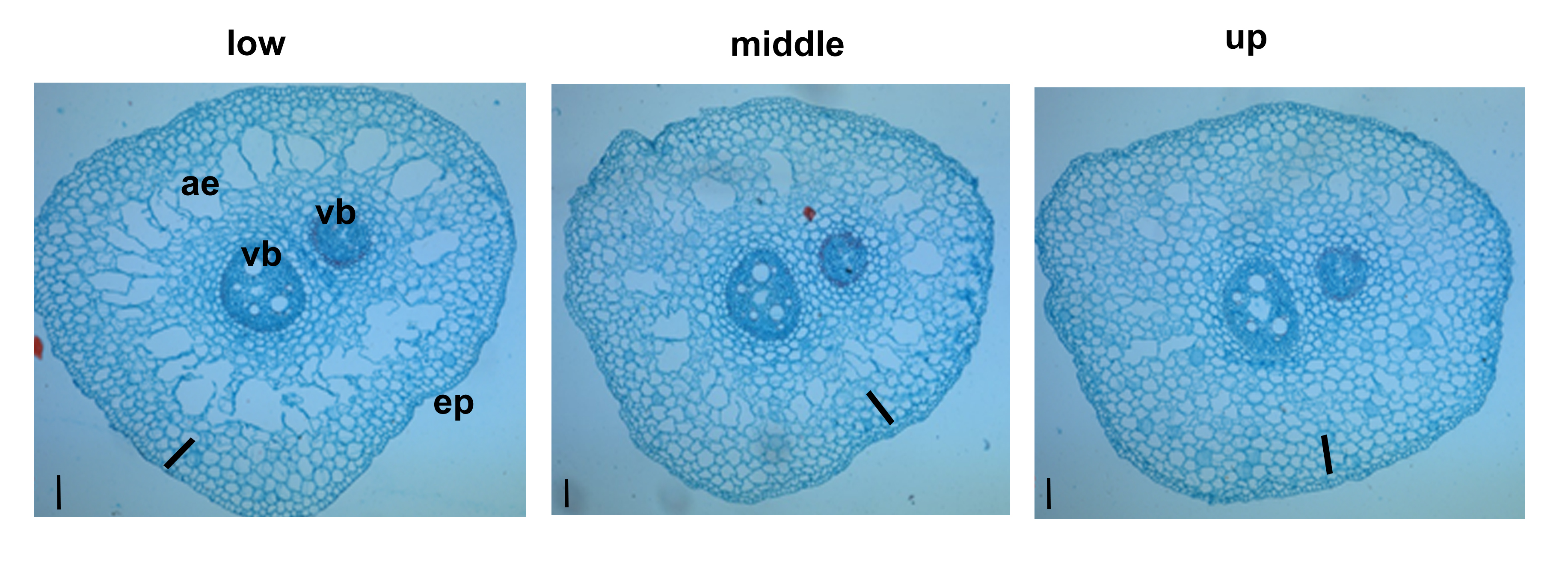


**Supplementary Figure 3. Cross sections from different parts of the mesocotyl.** The black lines indicate the cell layers used for cell number analysis. ae: aerenchyma; vb: vascular bundle; ep: epidermis. Scale bar, 50 µm.


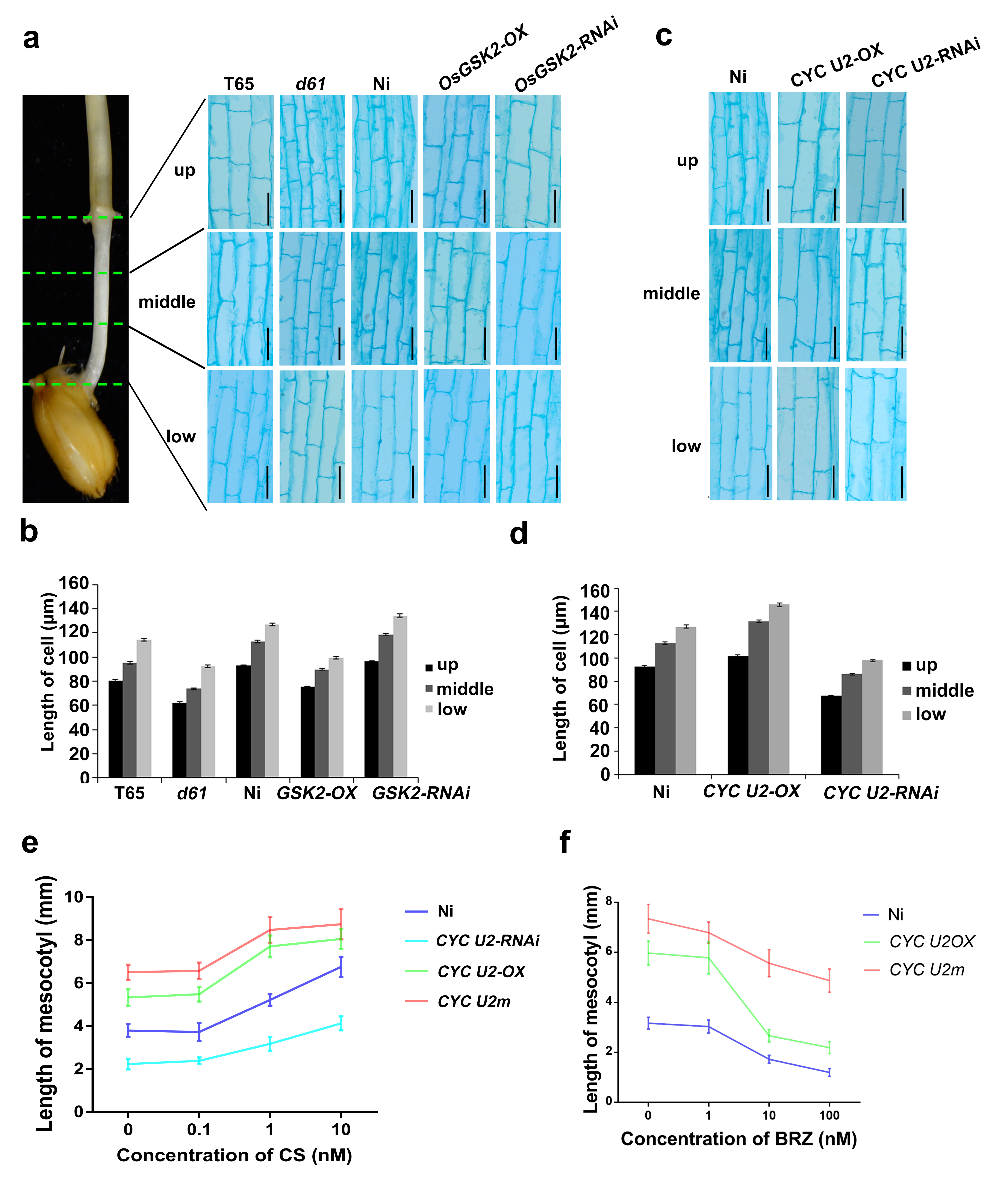


**Supplementary Figure 4. The phenotype of mesocotyls in the BR-related lines and *CYC U2* transgenic lines. (a)** Longitudinal sections of the mesocotyls in the BR-related lines. Scale bar, 50µm. **(b)** Quantification of cell length in mesocotyls shown in (a). Error bars are SE. (n =600). **(c)** Longitudinal sections of the mesocotyls in *CYC U2* tansgenic rice. Scale bar, 50µm. **(d)** Quantification of cell length in mesocotyls shown in (c). Error bars are SE. (n =600). **(e)** The mesocotyl length of the wild type and *CYC U2* transgenic lines responding to the different CS concentrations. The rice seedlings are grown on the medium with the indicated concentrations of CS for 7 days. Data are means ± SE (n = 30). **(f)** The mesocotyl length of the wild type and *CYC U2* transgenic lines responding to the different BRZ concentrations. The rice seedlings were grown on the medium with the indicated concentrations of BRZ for 7 days. Data are means ± SE (n = 30).


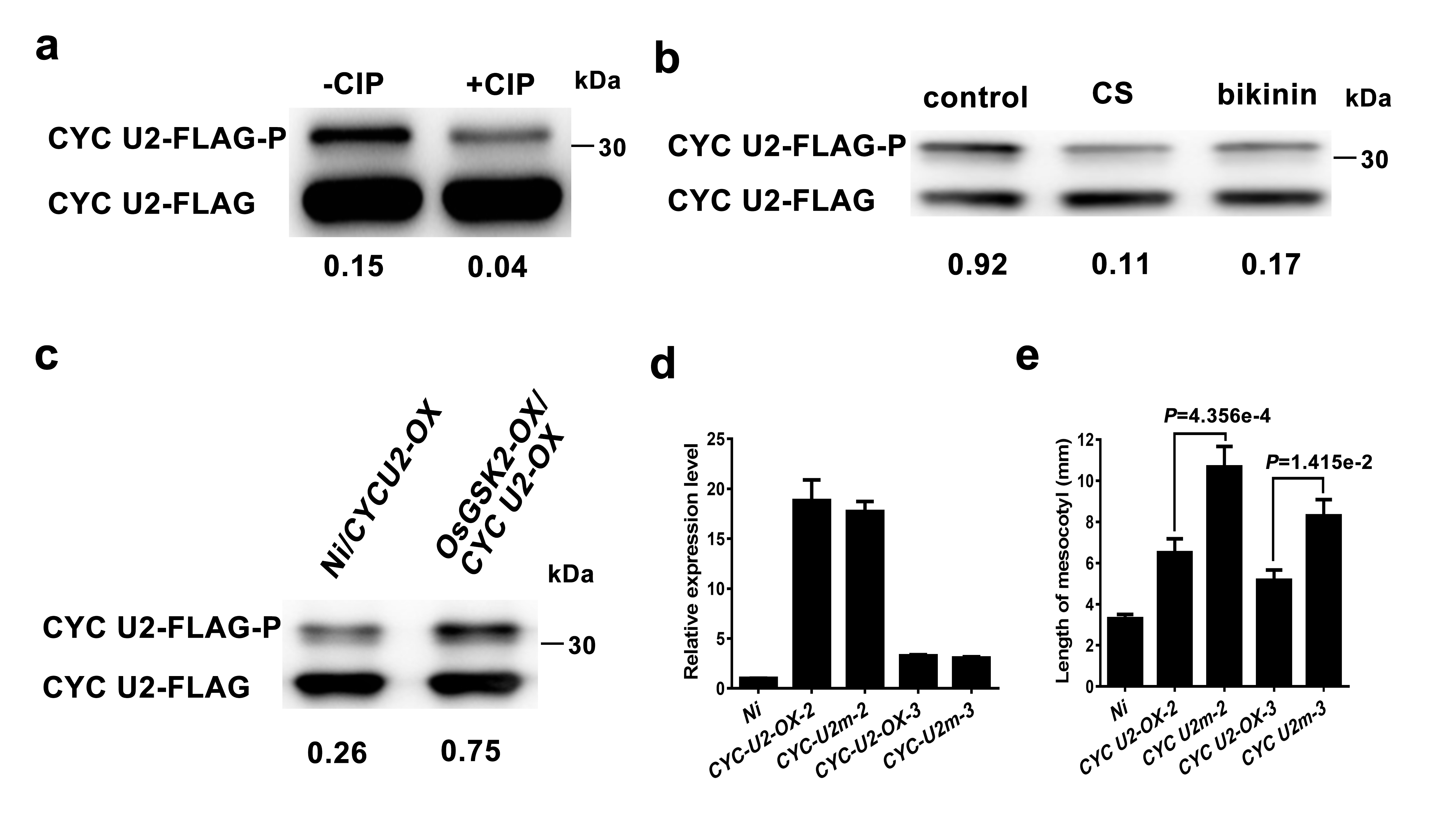


**Supplementary Figure 5. CYC U2 is phosphorylated by OsGSK2 in *planta.* (a)** The immunoprecipitated CYC U2-FLAG protein from the mesocotyls of *CYC U2-OX* plants was treated with or without CIP (as control) at 37°C for 1 h. The signal was detected by anti-FLAG. The numbers indicated the ratio of the phosphorylated CYC U2 to the unphosphorylated CYC U2. **(b)** The immunoprecipitated CYC U2-FLAG protein from the mesocotyls of *CYC U2-OX* plants grown on the medium containing DMSO (as control), 100 nM CS or 50 μM bikini for 5 days. The signal was detected by anti-FLAG. The numbers indicated the ratio of the phosphorylated CYC U2 to the unphosphorylated CYC U2. **(c)** The immunoprecipitated CYC U2-FLAG protein from the seedlings of Ni/*CYC U2-OX* or *OsGSK2-OX*/*CYC U2-OX* F_1_ hybrids lines. The signal was detected by anti-FLAG. The numbers indicated the ratio of the phosphorylated CYC U2 to the unphosphorylated CYC U2. **(d)** The expression levels of *CYC U2* in Ni, the independent transgenic lines *CYC U2-OX* and *CYC U2m*. Error bars are SD (n=3). **(e)** The mesocotyls length of the materials in (d) at the fifth day after germination. Error bars are SE (n=20). *P* values were determined by Welch’s *t*-test with Bonferroni correction.


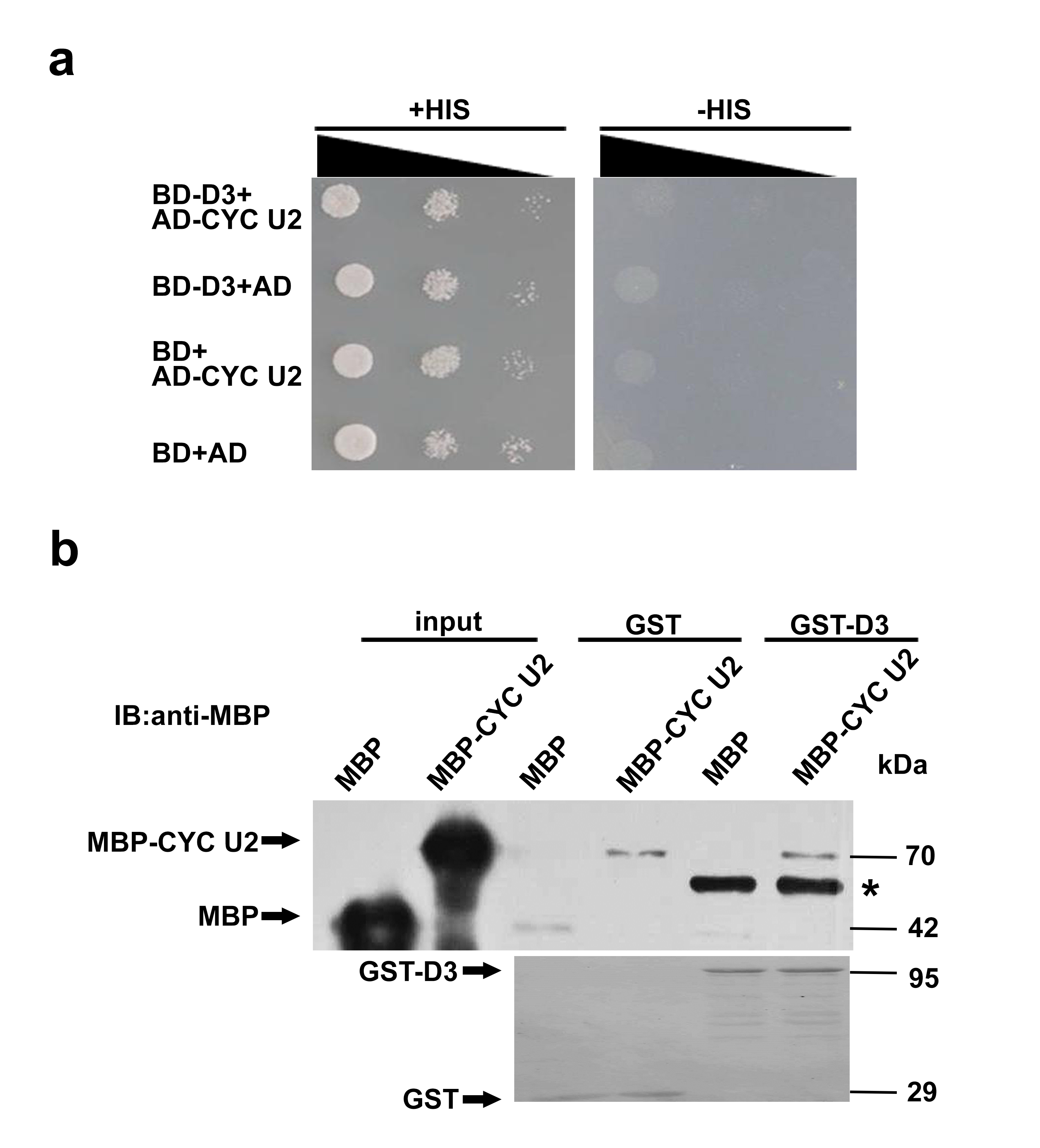


**Supplementary Figure 6. CYC U2 does not interact with D3 in vitro.** **(a)** CYC U2 does not interact with D3 in a yeast 2-hybrid assay. **(b)** CYC U2 does not interact with D3 in a GST-pull down assay. The bottom panel is the protein loading control. Asterisk indicates the nonspecific binds.


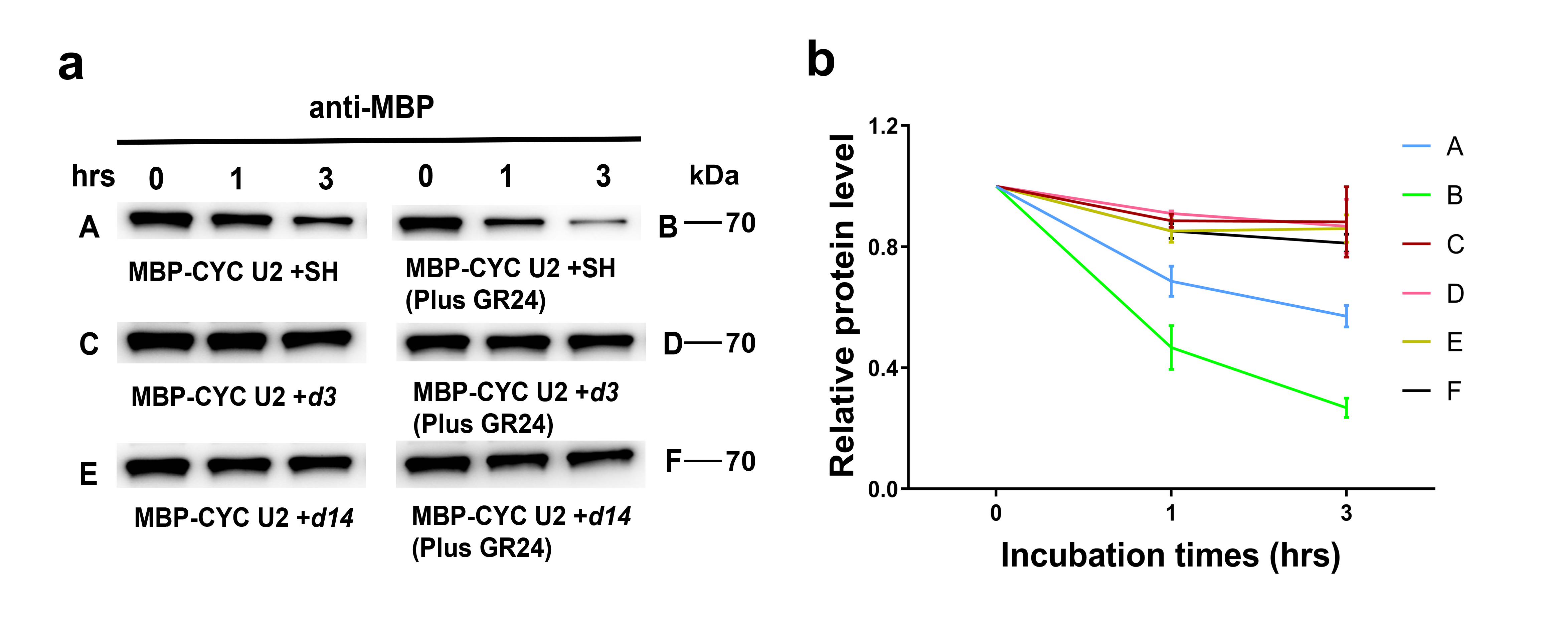


**Supplementary Figure 7. CYC U2 stability is regulated by SL signaling.** **(a)** The cell-free degradation assays for detecting degradation of CYC U2 in SH, *d3* and *d14* with or without GR24 treatment. MBP-CYC U2 was incubated with the OsGSK2 in kinase assay buffer for 2 hours before the degradation assays. The equal recombinant proteins were incubated with the equal plant crude extracts. **(b)** Quantification analysis for (a). The relative levels of MBP-CYC U2 incubated with the indicated plant extracts at 0 hour were defined as “1”. The degradation assay has been independently repeated for 3 times (means ± SD) (n = 3).


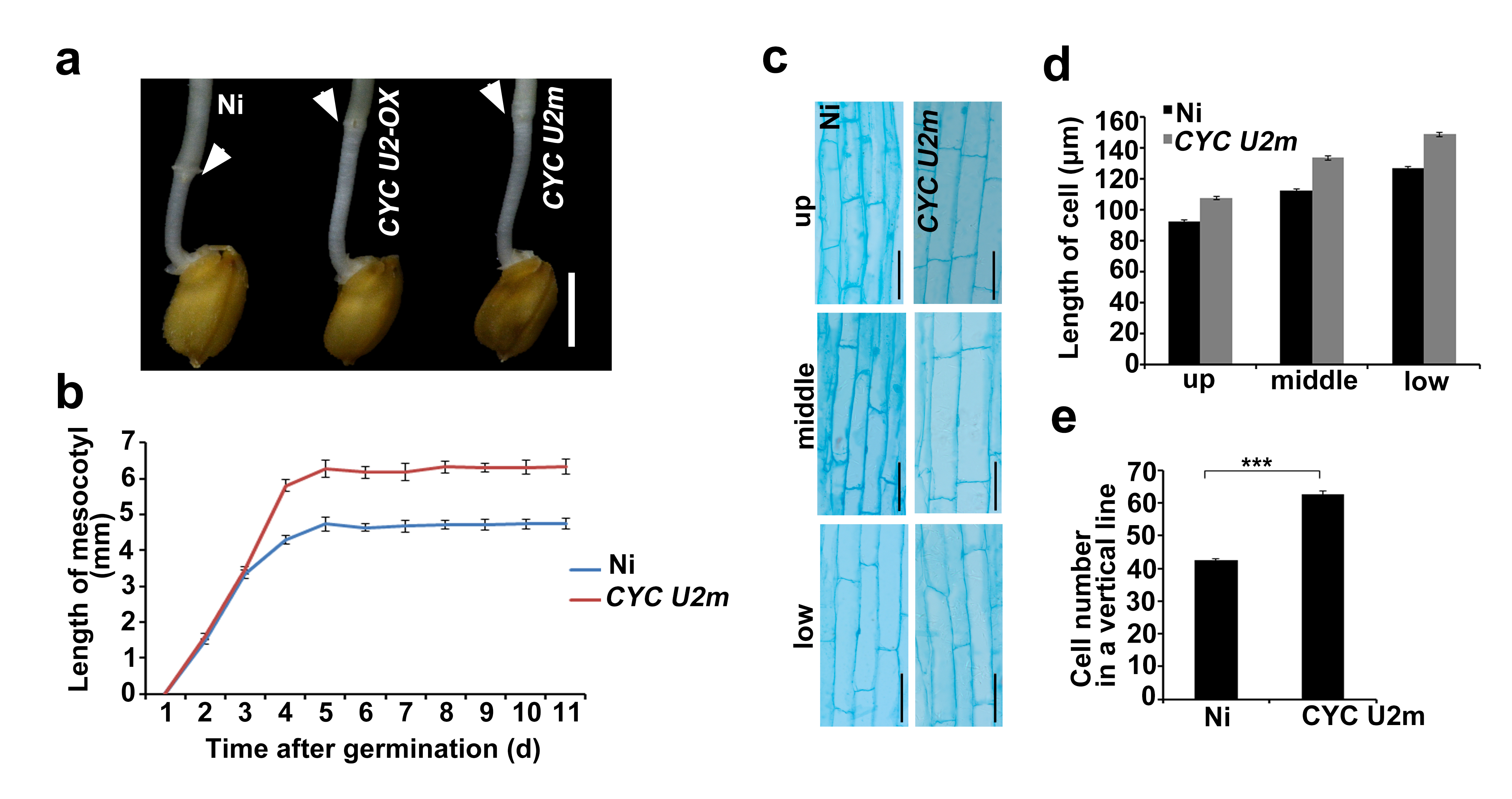


**Supplementary Figure 8. Phenotype of the *CYC U2m* transgenic rice. (a)** Mesocotyl morphology of Ni, *CYC U2-OX*, and *CYC U2m*. Arrows indicate the coleoptilar nodes. Scale bar, 0.5 cm. **(b)** Mesocotyl elongation rate in the *CYC U2m* transgenic lines at different time points. Ni (n=43), *CYC U2m* (n=35). **(c)** Longitudinal sections of mesocotyls from Ni and the *CYC U2m* transgenic lines. Scale bar, 50µm. **(d-e)** Cell length (n =420) (d) and cell number in an intact vertical line (e) (n =43) of mesocotyls from Ni and the *CYC U2m* transgenic lines.

Error bars are SE. *P* values were determined by Student's *t*-test. The significance level: *** P<0.001.


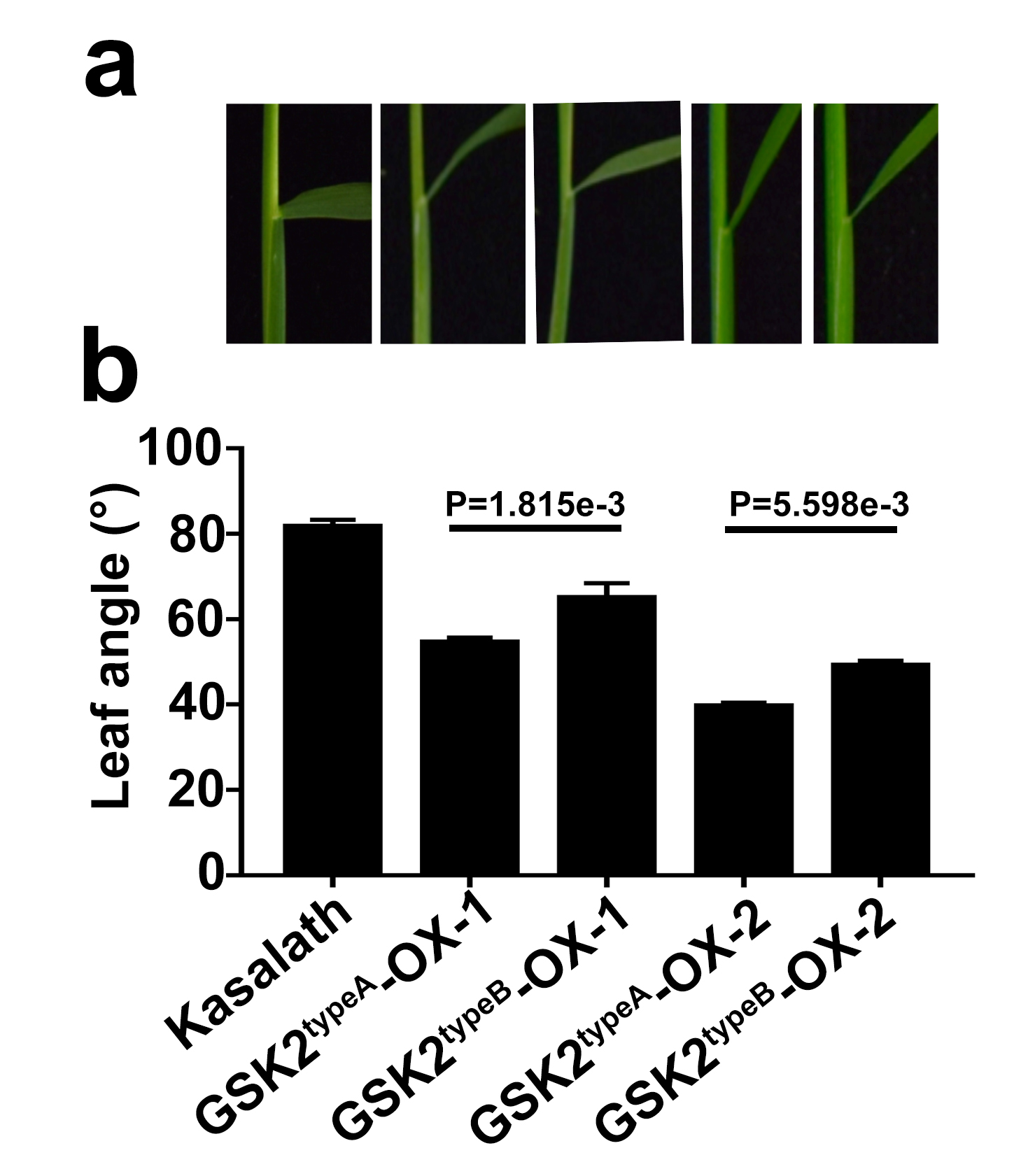


**Supplementary Figure 9. The leaf angle phenotype of the *OsGSK2^typeA^-OX* and *OsGSK2^typeB^-OX* transgenic rice. (a)** Morphology of leaf angles in the *OsGSK2^typeA^-OX* and *OsGSK2^typeB^-OX* transgenic lines in Kasalath background. Two-weeks old seedlings were used and the first complete leaf joints were showed. **(b)** The statistical data of leaf angle in (a). Kasalath is the wild type control. Error bars are SE (n=20). *P* values were determined by Welch’s *t*-test with Bonferroni correction.


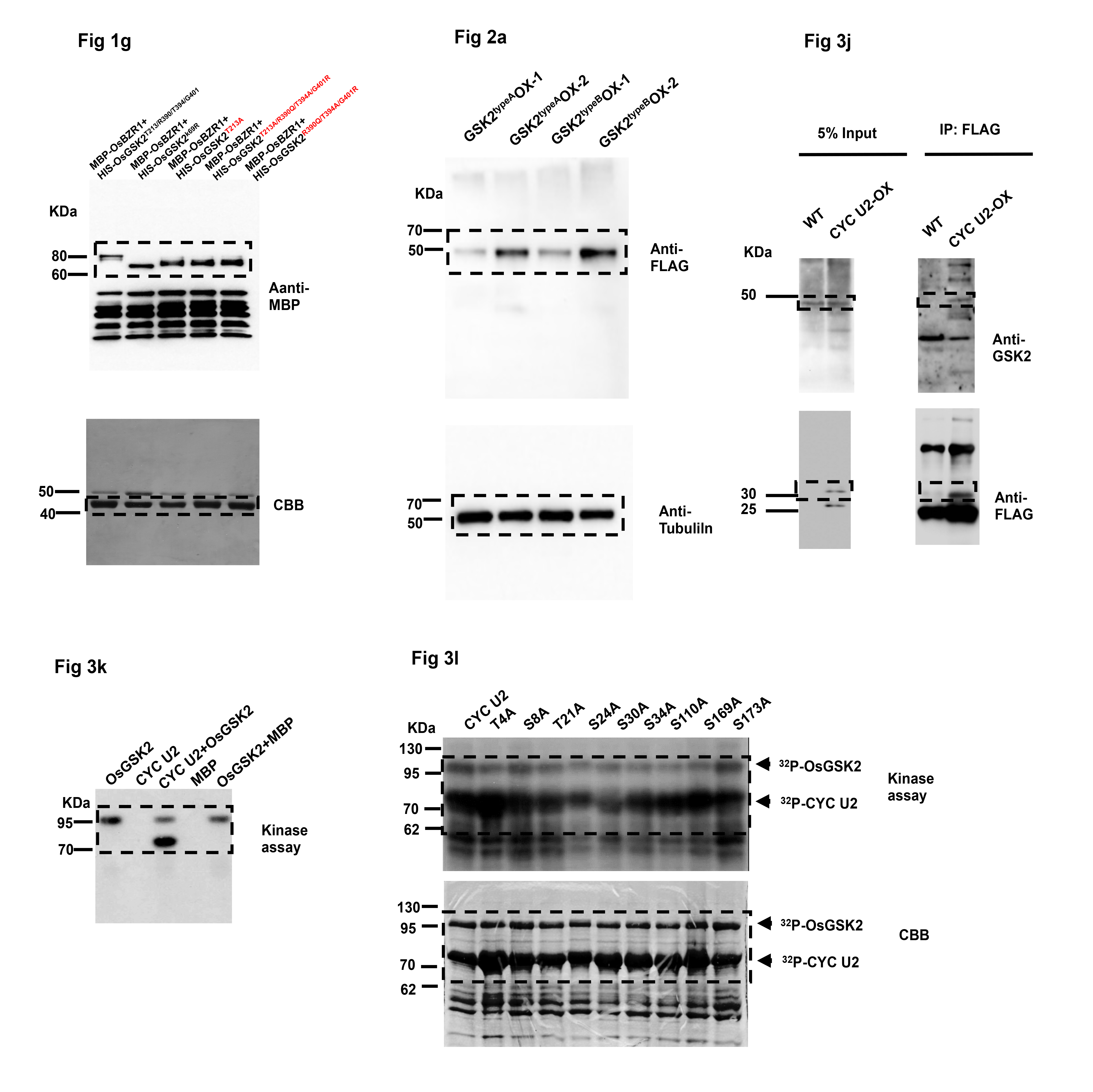


**Supplementary Figure 10. Uncropped images of blots shown in Fig. 1, 2 and 3.**


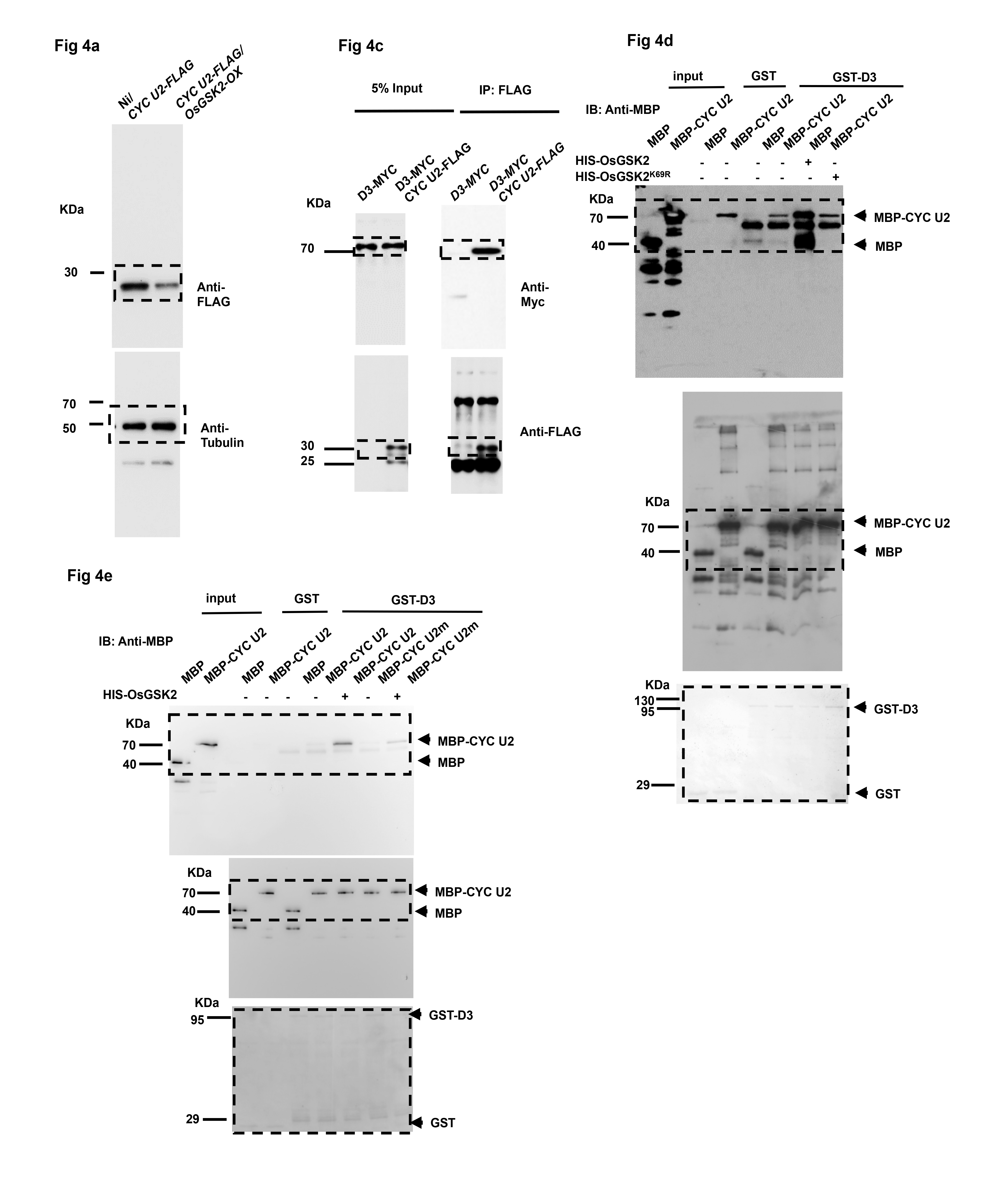


**Supplementary Figure 11. Uncropped images of blots shown in Fig.4.**


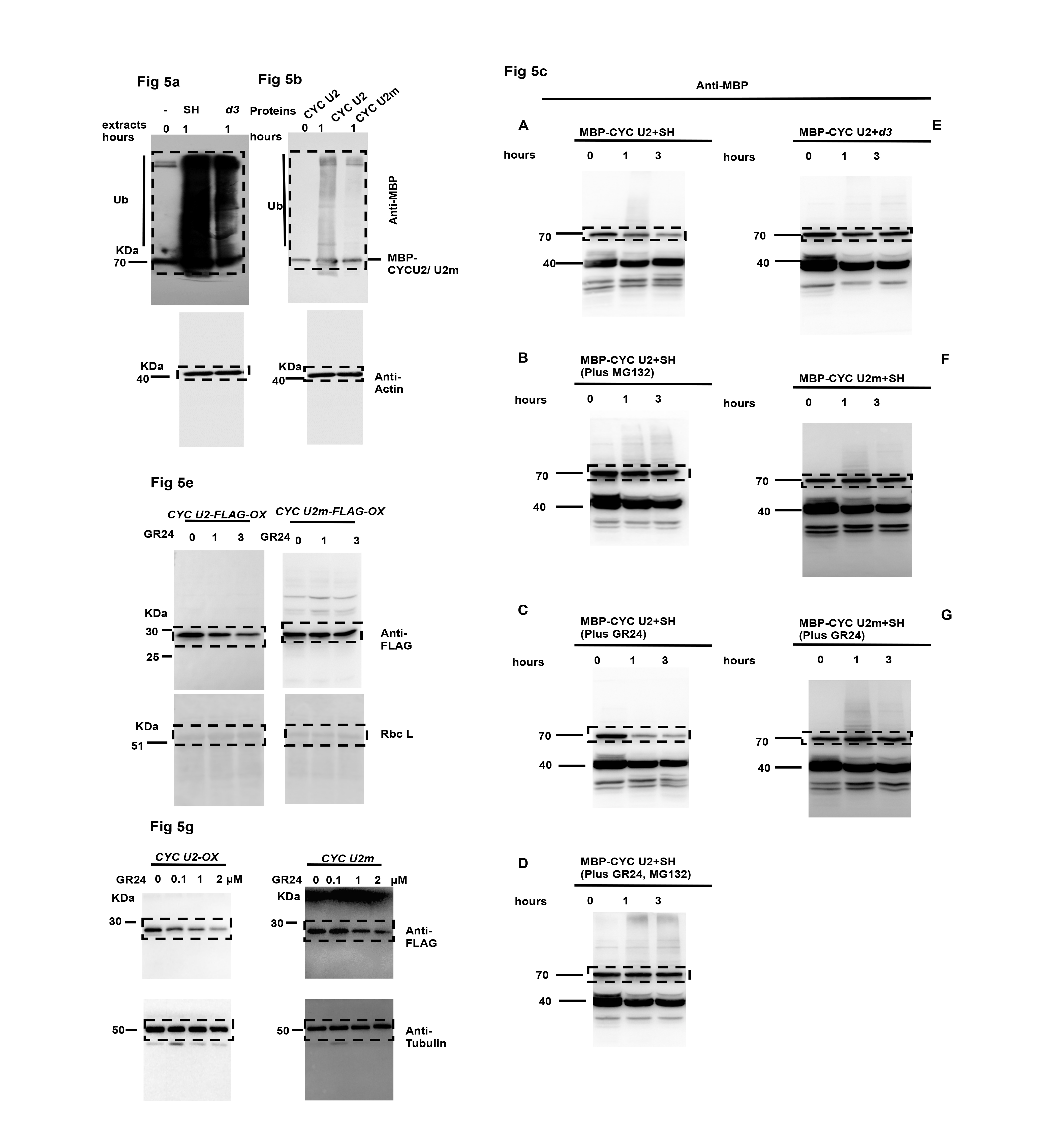


**Supplementary Figure 12. Uncropped images of blots shown in Fig.5.**


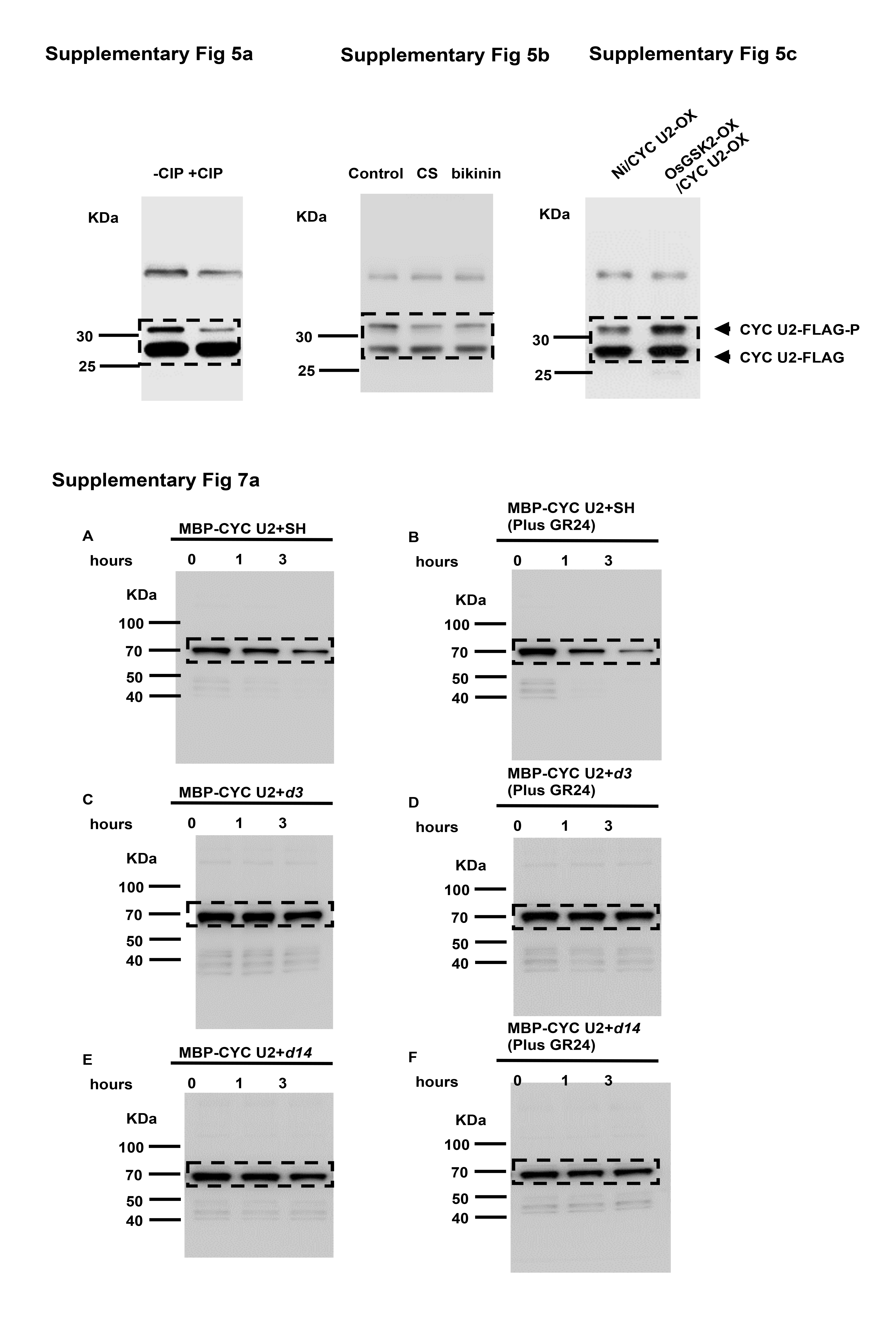


**Supplementary Figure 13. Uncropped images of blots shown in Supplementary Fig.5 and 7.**

**Supplementary Table 1. Genome-wide significant association signals of mesocotyl length.**


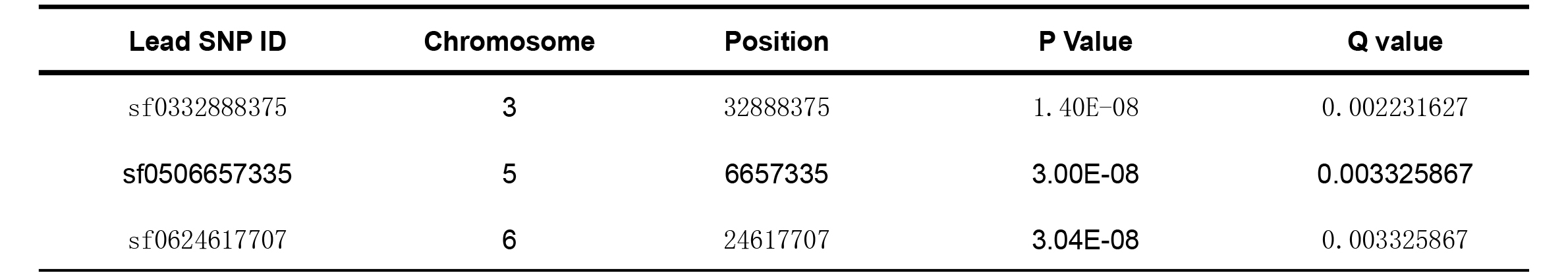


Q-value was used to measure False Discovery Rate (FDR) to determine *P* value, Q-value＜0.05 suggests that these *P* values were believable.

**Supplementary Table 2. Annotation of candidate genes anchored by the lead SNP on Chr 5 (±50Kb range) associated to the mesocotyl length.**


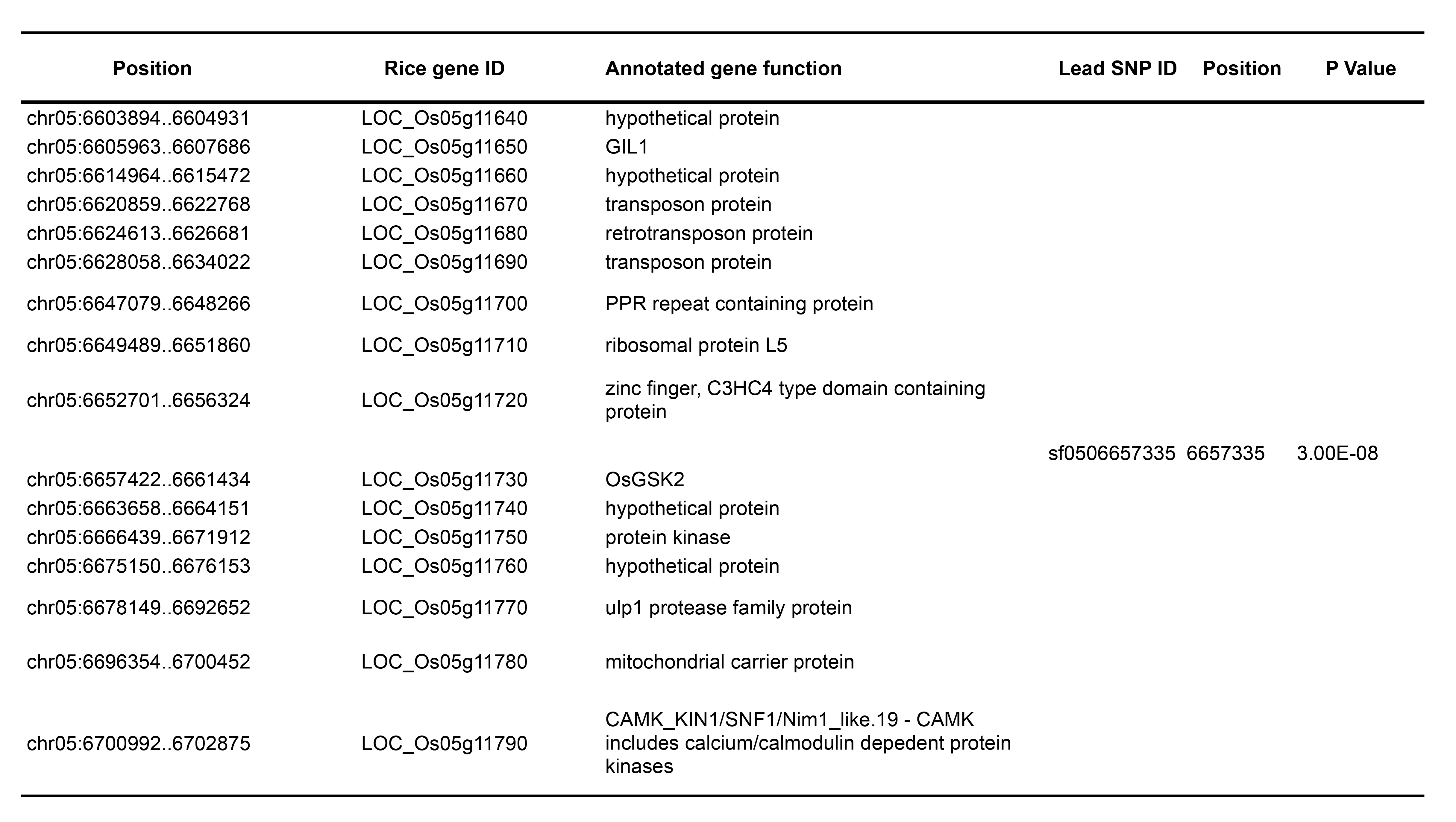

Supplement: Supplementary file 1 — Supplementary Information [file 41467_2018_4952_MOESM1_ESM.docx]
