# Supplementary material for: Natural selection of a GSK3 determines rice mesocotyl domestication by coordinating strigolactone and brassinosteroid signaling
Source: Nat Commun. 2018 Jun 28;9:2523. doi: 10.1038/s41467-018-04952-9 (PMC6023860; doi:10.1038/s41467-018-04952-9)
Supplement: Supplementary file 2 — Descriptions of Additional Supplementary Files [file 41467_2018_4952_MOESM2_ESM.pdf]

Descriptions of Additional Supplementary Files:

File Name: Supplementary Dataset 1
Description: List of 510 rice accessions used for association analysis.

File Name: Supplementary Dataset 2
Description: List of the rice accessions used for OsGSK2 transcription analysis.

File Name: Supplementary Dataset 3
Description: The sequences of all primers used in this study.
